# A Decoy Oligodeoxynucleotides Disturbing Forkhead Box O3 Mediated *ctnna2* Transcriptional Repression Prevents Postoperative Neurocognitive Disorder in Mice

**DOI:** 10.1111/cns.70454

**Published:** 2025-08-26

**Authors:** Zhixin Wu, Dongkun Xie, Jing Zhao, Jianshuai Zhao, Huiqing Liu, Dong Xing, Tingting Gu, Yaru Guo, Dan Wang, Zhihong Lu, Hailong Dong, Junlong Zhao, Jiao Deng

**Affiliations:** ^1^ Department of Anesthesiology and Perioperative Medicine, Xijing Hospital Air Force Medical University (The Fourth Military Medical University) Xi'an China; ^2^ Key Laboratory of Anesthesiology Air Force Medical University (The Fourth Military Medical University), Ministry of Education Xi'an China; ^3^ Shaanxi Provincial Clinical Research Center for Anesthesiology Medicine Xi'an China; ^4^ State Key Laboratory of Holistic Integrative Management of Gastrointestinal Cancers, Department of Medical Genetics and Developmental Biology Air Force Medical University (the Fourth Military Medical University) Xi'an China

**Keywords:** α‐N‐catenin, decoy oligodeoxynucleotides, Foxo3, postoperative neurocognitive disorder, rs12472215, Sirt1

## Abstract

**Background:**

Perioperative cognitive disorder (PND) affects up to 31% of surgical patients. Although clinical studies have identified a variety of risk factors, no effective prevention has been developed. From our previous cohort of PND patients, several single‐nucleotide polymorphism (SNP) sites on *ctnna2* were identified. The current study aims to decipher the role and regulatory mechanism of *ctnna2* in the PND model and to develop decoy oligodeoxynucleotides (decoy) for the possible prevention of PND.

**Methods:**

Both mice model (exploratory laparotomy+isoflurane) and the neuronal model (TNFα+isoflurane, T + I) for PND were used. Bioinformatic research was utilized to identify transcriptive active areas on *ctnna2*, *foxo3* sequence, and to predict possible transcriptional factors for regulation. Molecular biological techniques were used to decipher the regulatory mechanism and specific sites of the Sirt1‐foxo3‐ctnna2 axis in the development of PND. Finally, an decoy targeting the Foxo3‐ctnna2 interaction was designed and tested for effectiveness in PND.

**Results:**

Our results showed that the SNP rs12472215 is located at a newly defined enhancer region within the *ctnna2* intron that can be regulated by Foxo3 in the human genome. The rs12472215 A>T mutation potentiates Foxo3's transcriptive inhibitory effect on *ctnna2*. Experimental laparotomy in mice revealed that hippocampal Foxo3 upregulation and α‐N‐catenin reduction are involved in PND development. ChIP‐PCR deciphered two regulatory sites (R1 and R2) of Foxo3 on *ctnna2* in the mice that are strengthened by T + I. siAscl1 abolished the rescue effect of carbenoxolone (CBX, Foxo3‐specific inhibitor) on α‐N‐catenin expression in the T + I model, indicating that Foxo3 inhibits *ctnna2* transcription indirectly through Ascl1. Reduction of Sirt1 increased acetyl‐Foxo3, which enhanced its stability in PND. Sirt1 activation reduced Foxo3 expression, acetyl‐Foxo3 level, and rescued α‐N‐catenin expression in T + I stimulated neurons. More importantly, the new decoy disturbing Foxo3‐ctnna2 interaction effectively prevents α‐N‐catenin reduction, CA1 pyramidal neuron morphological change, electrophysiological dysfunction, and improves cognitive deficit in PND mice.

**Conclusions:**

These results provided a new revenue for identifying targets and developing interventions for PND. The decoy, due to its specificity and short acting time, merits further exploration for possible clinical use.

## Graphical abstract

Rs12472215 A>T mutation induces enhanced transcriptional inhibition of Foxo3 on *ctnna2* in humans. Alterations in the Sirt1‐Foxo3/Ascl1‐ctnna2 axis and reduction of α‐N‐catenin are involved in a mouse model of PND. A decoy that disturbs the interaction between Foxo3 and *ctnna2* prevents PND in mice (Created in BioRender. Deng, J. (2025), http://BioRender.com/js560r8).

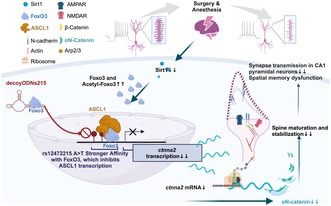

**Abbreviations**
AAV: Adeno‐Associated virus
ACSF: artificial cerebrospinal fluid
ASO: antisense oligonucleotides
CBX: carbenoxolone
ChIP‐qPCR: Chromatin Immunoprecipitation—Quantitative Polymerase Chain Reaction
Co‐IP: Co‐immunoprecipitation
CTCF: CCCTC‐binding factor
decoyODNs: decoy oligodeoxynucleotides
EPSC: excitatory postsynaptic current
GESA: Gene Set Enrichment Analysis
GWAS: Genome Wide Association Study
ODNs: oligodeoxynucleotides
PND: perioperative cognitive disorder
SNP: Single nucleotide polymorphism
TF: transcriptive factors
T + I: TNFα + isoflurane
TSS: transcription start site
WB: western blot

## 1 Introduction

Perioperative cognitive disorder (PND) is a common complication of anesthesia and surgery [1], with an incidence ranging from 1.9% to 31% [2, 3]. With more than 310 million surgeries conducted, PND can affect up to 5.89 to 65 million patients worldwide [4, 5] each year, this poses a significant challenge to patients' long‐term rehabilitation and leading to substantial wastage of medical resources. Although clinical studies have identified various risk factors associated with PND [6, 7], the pathogenesis remains unclear.

Patients undergoing the same anesthesia and surgery may or may not develop PND, suggesting potential internal factors influencing individual susceptibility to this condition. Single‐nucleotide polymorphisms (SNPs) may be involved in this phenomenon. Studies have unveiled that SNP polymorphisms such as CRP 1059C, SELP 1087A [8] and rs83 site on PDE4D [9] may be correlated with PND susceptibility. However, no study has explored how SNP mutations lead to increased susceptibility to PND or how to prevent or treat PND based on these mutations.

In our previous study [10] (*NCT02084030*), a Genome Wide Association Study (GWAS) revealed 28 SNP mutation sites with *p* < 1 × 10^−4^ in the peripheral blood cells of elderly patients who developed PND after on‐pump cardiac surgery, including up to 6 SNPs located at the *ctnna2* gene (encoding α‐N‐catenin) (see Figure S1). As a link between actin and β‐catenin, α‐N‐catenin plays a crucial role in cellular connectivity, cell morphology, and synaptic plasticity [11]. The *ctnna2* gene is a significant regulator of synaptic plasticity and has been reported to be involved in ADHD [12], schizophrenia [13, 14], obsessive‐compulsive disorder [15], and major depression [16]. Cummings et al. conducted a genome‐wide SNP linkage and association study on 798 individuals. Among 109 patients with late‐onset Alzheimer's disease, the most significant related SNP site, rs2974151, is located in the *ctnna2* gene [17]. Prokopenko et al. [18] also suggested that 13 genes, including *ctnna2*, *fnbp1l*, and *sel1l*, were new candidate risk gene loci for Alzheimer's disease [18]. These results pointed out that the *ctnna2* gene polymorphism may be involved in memory function after adulthood. Alteration of α‐N‐catenin expression, if it happened after surgery, may cause cognitive disturbance in the perioperative period. The primary aim of this study is to explore whether α‐N‐catenin expression is changed after surgery and if this change accounts for the cognitive decline in PND models.

Very few studies reported transcriptomic regulation of *ctnna2*. miRNA miR‐183‐5p and [19] miR‐4885 [20] were identified as possible regulators. But it is not known what or how transcriptive factors (TF) would regulate *ctnna2* in the setting of PND. Using bioinformatic prediction and experimental verification, the secondary aim of this study is to identify the transcriptomic regulatory mechanism of *ctnna2* in the PND model, and to test the role of the rs12472215 mutation in this mechanism.

A strategy targeting the interaction between transcription factor and the genome has been developed in cancer research called double‐stranded transcription factor decoy oligodeoxynucleotides (decoy). The decoy works as a competitor against the host's genome for TFs that have high affinity with the sequence, thus interfering with the TF's transcriptional regulatory effect. This technique provides therapeutic ODN candidates that specifically target and neutralize the effect of key TFs involved in the pathogenesis of a disease [21]. So finally, the third aim of this study is to design and verify the effect of a decoy that counteracts the influence of specific TF on *ctnna2* transcription and its in vivo neuroprotective effect in the PND model.

## 2 Methods

### 2.1 Animals

Male C57BL/6 mice aged 7–8 weeks and pregnant C57BL/6 female mice were provided by the Experimental Animal Center of the Fourth Military Medical University. All mice were housed under a 12‐h light/dark cycle (lights on from 7:00 to 19:00), 23°C ± 1°C temperature, 38% ~ 42% humidity, and free access to water and food. The experimental protocols were approved by the Ethics Committee for Animal Experimentation of the Fourth Military Medical University on March 4th, 2023. All the experiments were conducted according to the Guidelines for Animal Experimentation of the Fourth Military Medical University (Xi'an, China) and according to the ARRIVE guideline. For the behavioral study, 16–22 animals were used per group. Sample sizes in molecular biological experiments were chosen according to experience. Detailed sample sizes are listed in the statistical data file as Supporting Information.

### 2.2 PND Model in Mice and Cultured Neurons

#### 2.2.1 Animal Model

Experimental laparotomy was performed as previously described, with minor modifications [22]. Briefly, 8–11‐week‐old mice were anesthetized with 2.0% isoflurane (Baxter Healthcare, Puerto Rico, USA) through a breathing mask for 2 min, and anesthesia was maintained with 1.4% isoflurane. Mice were placed on a heating pad to maintain body temperature between 36.5°C and 37.0°C. A 1 cm vertical median incision was made 0.5 cm below the xiphoid. The right colon, diaphragmatic surface of the liver, spleen, kidneys, and bladder were explored with a sterilized cotton swab moistened with normal saline to mimic clinical exploratory laparotomy. Approximately 5 cm of the small intestine was gently pulled out and exposed to sterile gauze presoaked with normal saline. After being gently rubbed for 10 min, the intestine was returned to the abdominal cavity. The muscle and skin were closed layer by layer with 5–0 absorbable sutures (Polysorb, COVIDIEN, USA). Finally, 0.1 mL of 0.2% lidocaine was injected subcutaneously for postoperative analgesia. The total duration of anesthesia was 2 h. The mice were allowed to recover in a warm chamber for 30 min before being returned to their home cages.

#### 2.2.2 In Vitro Model of PND (With TNF‐α + Isoflurane Stimulation) and Drug Delivery

Pregnant C57BL/6 female mice were deeply anesthetized with pentobarbital sodium (70 mg kg‐1) and sacrificed on days 14 to 16 of pregnancy. The brain tissue block containing the cortices and the hippocampus was quickly dissected from the embryos. After trypsinization, a single‐cell suspension was obtained and resuspended in neurobasal medium (Gibco, 21103049) supplemented with 2% B27 (Gibco, 17504‐044), 1% penicillin/streptomycin (HyClone, SV30010), and 1% L‐glutamate (Gibco, 35050‐038). The neurons (1 × 10^6^) were cultured in six‐well plates precoated with poly‐D‐lysine (50 μg mL^−1^) at 37°C and 5% CO_2_. The media were changed every 3 days. At 6 days in vitro, neurons were treated with TNF‐α (40 ng mL^−1^) (CST, #16769) and isoflurane (1.4%, carried by 21% O_2_ and 5% CO_2_) (T + I) for 2 h. At 7 days in vitro, neurons were collected for Western blot (WB) or Chromatin Immunoprecipitation‐Quantitative Polymerase Chain Reaction (ChIP‐qPCR) experiments.

SH‐SY5Y cells were purchased from Pri‐cella (Wuhan, China). Neurons were differentiated with 5 days of retinoic acid, followed by 7 days of BDNF, as previously described [23] before use. The in vitro model mimicked anesthesia and surgery and was similar to that of primary cortical neurons.

For the Ascl1 siRNA experiment, neurons were transfected with Ascl1‐siRNA according to the manufacturer's protocol (sc37693, Santa Cruz Biotechnology, Dallas, TX) using the Lipofectamine protocol (Invitrogen, Thermo Fisher Scientific, US) [24]. After 48 h of Ascl1 silencing, neurons were treated with T + I stimulation. Twenty‐four hours later, cells were harvested and homogenized for western blot analysis.

For the SirT1 activation experiment, SRT1720 (S1129, Selleckchem, China) was added to the cell culture medium to make a final concentration of 1.5 μM and incubated with neurons for 2 h before the T + I stimulation.

For the carbenoxolone (CBX)rAAV‐hSyn‐mCherry‐shCTNNA2, rAAV‐hSyn‐mCherry‐Scramble, rAAV‐hSyn‐CTNNA2‐mCherry, or rAAV‐hSyn‐mCherry experiment, CBX solution was added to the culture medium to make a final concentration of 100 μM and incubated with neurons for 2 days before the T + I stimulation.

### 2.3 Barnes Maze Test

Seven days after surgery, the Barnes maze was used to test the spatial learning and memory of mice as previously described [21]. Animals were allowed to acclimate to the behavioral testing room for 30 min each day during training and testing. Briefly, the animals were randomly assigned to different groups before the experiment. After 4 consecutive days of the training phase (3 min per trial, 2 trials each day for 4 days and 2 h interval between trials), the spatial memory test was conducted 1 day after training (short‐term retention) and 8 days after training (long‐term retention); the escape compartment was removed, and mice were allowed to move freely for 2 min. All sessions were videotaped and analyzed blindly. The latency to enter the escape quadrant and the percentage of time spent in the target quadrant in 2 min were calculated to assess the spatial reference learning and memory ability of the mice. All data were analyzed with Anymaze (Stoelting, San Diego, USA).

### 2.4 Adeno‐Associated Virus (AAV) and CA1 Injection

Mice aged 7–8 weeks were anesthetized with 1.4% isoflurane and secured on a stereotaxic apparatus. The skin on top of the head was shaved and sterilized, a small central incision was made, and the fascia was removed. After the hippocampus was located, a hole was drilled through the skull, and 150 nL of 2 × 10^12^ vg ml^−1^ (brainvta, Wuhan, China) was bilaterally delivered into the dorsal hippocampus (AP: − 2.0 mm; ML: ± 1.40 mm; DV: − 1.6 mm) at a speed of 50 nL min^‐1^. The shCTNNA2 target sequence was as follows: GGATTTATTAGCCTACCTTCA. The syringe was left in position for an additional 10 min. After injection, the incision was carefully sutured, and the mice were allowed to recover on a heating pad before being returned to their home cages. Laparotomy was performed 3 weeks later.

### 2.5 Intracerebralventricular Injection of Carbenoxolone

Mice were anesthetized by intraperitoneal injection of sodium pentobarbital (50 mg kg^−1^, i.p.) and secured on a stereotactic frame with a heating pad to maintain rectal temperature at 36°C–37°C. The coordinates for the left cerebral ventricle cannula tip were set at (AP: − 0.50 mm; ML: + 1.00 mm; DV: − 2.0 mm). Stainless steel guide cannulas (length: 3 mm, outer diameter: 0.65 mm) were implanted at the coordinates mentioned above and stabilized with dental cement and 2 anchoring screws. For post‐surgical analgesia, mice were subcutaneously injected with 0.1 mL of 0.2% lidocaine. The animals were allowed to recover for 7 days before intracerebroventricular injection of CBX.

A specific Foxo3 inhibitor, CBX (Sigma‐Aldrich, C4790‐1G) was dissolved in artificial cerebrospinal fluid (ACSF) at a final volume of 500 nL and delivered with a micropump at 100 nL min^−1^ into the left lateral cerebral ventricle through the cannula in freely moving mice once at 30 min before surgery and again at 3 days after surgery. Dosage was set at 50 μg kg^−1^ or 100 μg kg^−1^. For the control group, a cannula was implanted; animals were anesthetized without CBX medication.

### 2.6 Immunofluorescent Staining and Golgi Staining

#### 2.6.1 Immunofluorescent Staining

Mice were deeply anesthetized with sodium pentobarbital (70 mg kg^−1^, i.p.) and transcardially perfused with 0.9% saline followed by 4% paraformaldehyde in PBS 3 days after surgery and anesthesia (or control condition). The brains were harvested, postfixed in 4% paraformaldehyde overnight at 4°C, and dehydrated in 30% sucrose in PBS for cryoprotection. A freezing microtome (Leica, Germany) was used to obtain 15 μm coronal slices. The sections were incubated with 0.3% Triton X‐100 in PBS for 10 min, blocked with 10% donkey serum for 30 min, and then washed with PBS. For double immunofluorescence staining, the sections were incubated with a mixture of anti‐α‐N‐catenin (CST, ab24182, 1:200, rabbit) and anti‐MAP2 (arigobio, ARG52328, 1:200, Chicken) antibodies overnight at 4°C. After three rinses, the sections were incubated with a mixture of Alexa 594‐conjugated donkey–anti‐rabbit IgG (Invitrogen, 1:400) and Alexa 488‐conjugated donkey–anti‐chicken IgG (Invitrogen, 1:400) antibodies for 2 h at room temperature. The slides were washed and cover‐slipped with mounting media containing DAPI (Abcam, ab228549, 1:10000) for observation under a confocal laser scanning microscope (FV10, Olympus, Japan).

#### 2.6.2 Golgi Staining

Mice brains were dissected and immersed in Golgi staining solution, which contained 50% potassium dichromate (MP Biomedical, 021563389), 5% mercuric chloride (Sigma‐Aldrich M1136), and 5% potassium chromate (Sigma‐Aldrich 529508) and protected from light for 7 days. When performing Golgi staining, brain sections were washed with distilled water, dehydrated with ethanol, and then treated with ammonia (3:1). The sections were subsequently washed and incubated in 5% sodium thiosulfate for 10 min and then dehydrated with graded ethanol and clarified with xylene. The stainings were observed under the bright field of an FV1000 microscope (Olympus, Japan). Five to ten pyramidal neurons were chosen randomly from the corresponding region in the CA1 of the hippocampus in each group, and at least two dendritic segments were used for analysis. The quantification of the densities of total and various categories of dendrite spines was performed manually using the criteria established earlier [25].

### 2.7 Western Blot Analysis

Mice were deeply anesthetized with sodium pentobarbital (70 mg kg^−1^, i.p.) and transcardially perfused with 0.9% saline. Hippocampal tissues were harvested and homogenized in RIPA lysis buffer comprising 1 mM phosphatase inhibitor, 1 mM protease inhibitor (Roche Applied Science, Basel, Switzerland), and 1 mM phenylmethylsulfonyl fluoride (PMSF, Beyotime Biotechnology, Shanghai, China) with an ultrasonic processor. The supernatant was collected, loading buffer was added, and the mixture was boiled for 10 min. The proteins were separated by SDS‐PAGE (Cowin Biosciences, Beijing, China) and then transferred to a methanol‐soaked polyvinylidene fluoride membrane (GE Healthcare, Boston, USA). The membrane was blocked with 5% milk (BD, New Jersey, USA) for 1 h at room temperature and then incubated with primary antibodies overnight at 4°C, followed by HRP‐conjugated secondary antibodies for 1 h at room temperature. The following primary antibodies were used in this study: anti‐α‐N‐catenin (CST, #2131, 1: 1000, rabbit), anti‐SIRT1 (Abcam, ab110304, 1:1000, mouse), anti‐Acetyl‐FOXO3A (lys271) (Affinity Biosciences, AF3771, 1:500, rabbit), anti‐FoxO3a (CST, #99199, 1:1000, mouse), anti‐FoxO3a (Proteintech, 10849‐1‐AP, 1:1000, rabbit), anti‐β‐Actin (Proteintech, 20536‐1‐AP, 1:1000, rabbit), anti‐β‐Tubulin (Abbkine, ABL1030, 1:2000, mouse). The results were analyzed with Image Lab software (Bio‐Rad, Hercules, CA, USA).

### 2.8 Quantitative Real‐Time Polymerase Chain Reaction (PCR)

After anesthesia with sodium pentobarbital (70 mg kg‐1, i.p.), mice were subjected to intracardiac perfusion with 0.9% refrigerated saline. The hippocampus was harvested, frozen in liquid nitrogen, and kept at −80°C. Total mRNA was extracted from tissues using the TRIzol reagent (Invitrogen) and the Total RNA Extraction Kit (TIANGEN Biotech). The concentration of the extracted RNA was measured with a spectrophotometer. Quantitative real‐time PCR (qRT‐PCR) was performed with PrimeScript RT Master Mix (TaKaRa) according to the manufacturer's protocol. Each cDNA sample was denatured at 95°C for 5 min and amplified for 35 cycles of 15 s at 98°C, 30 s at 58°C, and 30 s at 72°C with a LightCycler 96 (Roche). The mRNA expression level of each target gene was normalized to GAPDH and analyzed using LightCycler 96 Application Software (Roche). The qRT‐PCR primers are listed in Table S1.

### 2.9 Electrophysiology for CA1 Pyramidal Neuron in Hippocampal Slices

The brain was removed from mice (6–8 weeks old) and kept in pre‐oxygenated ice‐cold solution containing (in mM) 124 NaCl, 25 NaHCO_3_, 2.5 KCl, 1 NaH_2_PO_4_, 2 CaCl_2_, 2 MgSO_4_, and 37 glucose, saturated with 95.0% O_2_ and 5.0% CO_2_. Brain slices (300 μm) were cut using a vibratome (Leica VT 1200 s, Germany). Slices were then transferred to a chamber with ACSF containing (in mM) 124 NaCl, 25 NaHCO_3_, 2.5 KCl, 2 CaCl_2_, 1 NaH_2_PO_4_, 1 MgSO_4_, and 10 glucose and saturated with 95.0% O_2_ and 5.0% CO_2_. Slices were incubated at room temperature for 1 h before patch clamp recording.

The recordings were performed with an Axon 700B amplifier (Molecular Devices, USA). Clampex (Molecular Devices) was used to acquire the data. The whole‐cell patch‐clamp recordings were made from the pyramidal neurons of the CA1 region in voltage‐clamp mode. For sEPSC recording, the recording pipettes (3–5 MΩ) were filled with an internal solution containing (in mM): 124 K‐gluconate, 5 NaCl, 1 MgCl_2_, 0.2 EGTA, 2 MgATP, 0.1 Na_3_GTP, 10 HEPES, and 10 phosphocreatine disodium (adjusted to pH 7.2 with KOH, 290 mOsmol). For AMPA/NMDA current recordings, the internal solution contained (in mM): 112 Cs‐gluconate, 5 TEA‐Cl, 3.7 NaCl, 0.2 EGTA, 10 HEPES, 2 MgATP, 0.3 Na_3_GTP, and 5 QX‐314 (adjusted to pH 7.2 with CsOH, 290 mOsmol). AMPAR‐mediated current was recorded at −70 mV. NMDA‐R‐mediated current was recorded at +40 mV. All experiments were performed with picrotoxin (Sigma‐Aldrich, 100 μM) bath application. Data were discarded if the access resistance changed > 15% during the experiment. Data were filtered at 1 kHz and digitized at 10 kHz. All whole‐cell patch data analyses were performed with the Mini Analysis Program (Synaptosoft, USA) and Clampfit 10.2.

### 2.10 Gene Sequencing of SH‐SY5Y Cells

Gene sequencing was performed using Sanger sequencing by Genechem Co. Ltd. (Shanghai, China).

### 2.11 Reporter Assay

The reporter assay was performed using a commercial kit (Invitrogen, Carlsbad, CA). The enhanced sequence near the rs12472215 site was inserted into the pGL3‐promoter plasmid. HEK 293 T cells were inoculated into 96‐well plates, and Lipofectamine 2000 (0.5 μL) and pGL3‐rs12472215 promoter (0.1 μL) or empty plasmid (0.1 μL) were added to each well, while different concentrations of the target gene overexpression plasmid and the internal reference gene TK plasmid were added for transfection, and the cells were switched to a complete culture medium containing 10% FBS after 6 h of transfection, and cultured for 48 h. The reporter genes were detected using the reporter gene kits (Invitrogen, 16181, Carlsbad, CA).

### 2.12 ChIP‐PCR

The ChIP assay was performed using a kit from Millipore (Merck Millipore, Billerica, MA). Primary mouse cortical neurons with different treatments were fixed with 1% formaldehyde for 10 min to cross‐link the proteins with the chromatin. The cross‐linking was terminated by adding glycine at a final concentration of 0.125 M (5 min). The treated cells were obtained and lysed with SDS Lysis buffer. Harvested cells were sonicated to break the cross‐linked protein‐DNA complexes into fragments. The supernatant was harvested, and primary antibodies were added to form immune complexes with the target protein. The immune complexes were washed and eluted using wash buffer and elution buffer, and the cross‐linking was reversed by adding NaCl at a final concentration of 0.2 M (65°C, 6 h). The DNA fragments obtained were purified by spin columns, and the purified DNA samples were analyzed by PCR. The following primary antibodies were used in this study: FOXO3A Antibody (Invitrogen, #720128, rabbit), SIRT1 Antibody (Invitrogen, #703653, rabbit). The ChIP‐PCR primers are listed in Table S1.

### 2.13 Chromosome Conformation Capture (3C)

Two groups of primary mouse cortical neurons were used. One control group and another group were treated with the universal Fox inhibitor FoxO1‐IN‐3 (20 μM, MedChemExpress, Nashville, TN) to test whether Fox affected the chromosome interaction of the *ctnna2* gene in mice. Cells were treated with 1% formaldehyde to cross‐link chromatin, immobilize cellular proteins and DNA, and maintain the three‐dimensional structure of chromatin. Chromatin was cleaved with the restriction endonuclease HindIII (Thermo Scientific, Waltham, MA) to separate target protein‐interacting genes from non‐interacting genes. Interacting DNA fragments were ligated into loops, and the cross‐links were reversed by incubation at 65°C for 5 h. The interaction between the regulatory elements was validated using PCR. The primers are listed in Table S1.

### 2.14 Design, Synthesis, and Sequencing of Conjugated Decoy Oligodeoxynucleotides (Decoy)

Conjugated decoy oligodeoxynucleotides were synthesized by AuGCT (Beijing, China), primarily by the solid‐phase phosphorimide triester method, using the ABI391 synthesizer (Applied Biosystems, Carlsbad, CA). The products were then purified by the PAGE cutting method.

### 2.15 Delivery of Decoy Oligonucleotides

In the in vitro experiments, 0.5 nM of decoy in 1 μL of neural basal medium or its vehicle control was added to the 2 mL medium of 6‐well plates before T + I stimulation. After 3 h of co‐culture, the culture medium was changed.

In the in vivo experiment, 1 OD of the decoy or control DNA was injected intravenously into the tail vein with (1.6 nmol diluted in 160 μL of ddH_2_O) at the end of the surgery. Sequences were shown below:

**Table:** 

Decoy	TGTTCTGTAAACCCTAGTTCTGTAAACC TATTTTTTATAGGTTTACAGAA CTAGGGTTTACAGAACA
Control	TGTCAACTGTACTCTAGTCAACTGTACT TATTTTTTATAAGTACAGTTGA CTAGAGTACAGTTGACA

### 2.16 Statistical Analysis

All tests were performed with PRISM 10 or SPSS Version 18 software (SPSS Inc., Chicago, IL). Normality testing for all measured parameters was performed using the Shapiro–Wilk test. For comparisons between two groups, an unpaired t‐test was used. For non‐normally distributed data, the Mann–Whitney U test was utilized. For comparisons involving three or more unpaired groups, one‐way ANOVA with Tukey's post hoc test was conducted; if the Brown‐Forsythe test indicated unequal variances, Brown‐Forsythe ANOVA with Games‐Howell post hoc test was employed. For non‐normally distributed data across three or more groups, the Kruskal‐Wallis test with Dunn's post hoc test was used. When analyzing two variables, two‐way ANOVA followed by Sidak's multiple comparisons test was performed.

The significance level accepted for all tests was 0.05 with a 95% confidence interval. All values included in the figure legends are represented as mean ± SEM. All analyzed statistical data are provided in Data S1. Data are also deposited at the Mendeley website and ready to download: https://data.mendeley.com/datasets/8j6dn4gdm8/1.

### 2.17 Role of the Funding Source

The funding source of this study had no involvement in study design; in the collection, analysis, and interpretation of data; in the writing of the report; or in the decision to submit the paper for publication.

## 3 Results

### 3.1 Foxo3 Mediates *CTNNA2* Transcriptional Repression by Binding to a Newly Defined Enhancer Region Around rs12472215 in the Human Genome

**FIGURE 1:**
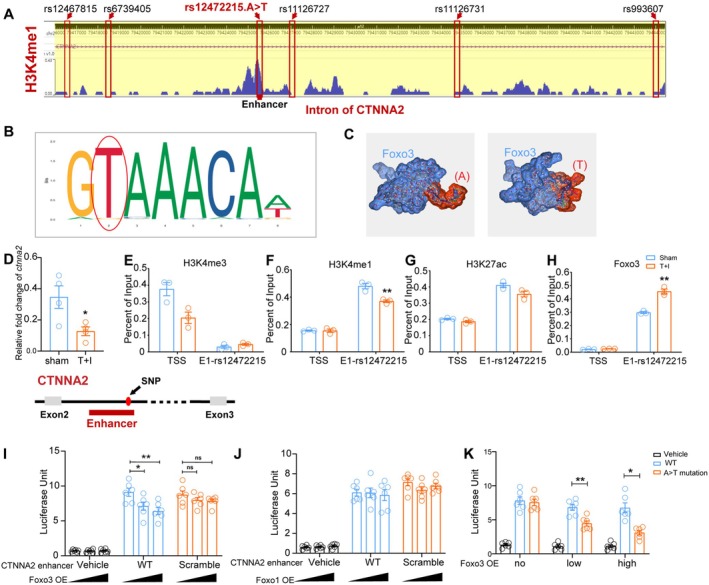
The *CTNNA2* transcription is inhibited by Foxo3 at a newly defined enhancer region near rs12472215, and this inhibition is stronger on rs12472215 (T) than on rs12472215 (A). (A) ChIP data from Davis et al. [26] showing that the region near rs12472215 in the intron of *ctnna2* on the human genome has a high affinity to H3K4me1. Red squares represent the 6 SNPs on *CTNNA2* that are differentially distributed between PND and non‐PND patients in the NCT02084030 cohort. (B) Jaspar's prediction of the sequence that Foxo3 binds to near rs12472215. The red oval indicates that the mutated T is predicted to have a higher affinity as compared to other bases (https://jaspar.genereg.net/analysis). (C) Haddock Prediction for spatial construction of Foxo3 protein (blue) and the 10 bp near rs12472215 (red). Left showing Foxo3 and rs12472215 (A), right showing Foxo3 and rs12472215 (T) (https://wenmr.science.uu.nl/haddock2.4/). (D) *CTNNA2* mRNA level in differentiated SH‐SY5Y cells treated w/o TNF‐α + iso stimulation. (E–H) DNA binding Affinity of H3K4me3 (F), H3K4me1 (J), H3K27ac (H), and Foxo3 (I) on the TSS and E1‐rs12472215 sequences in differentiated SH‐SY5Y neurons w/o TNF‐α + iso stimulation. (I) Reporter assay showing the effect of different transcripts of Foxo3 on luciferase intensity of the *CTNNA2* enhancer region sequence (WT) as compared to a scrambled sequence. (J) Reporter assay showing the effect of different transcripts of Foxo1 on luciferase intensity of the *ctnna2* enhancer region sequence (WT) as compared to a scrambled sequence. (K) Reporter assay showing effect of Foxo3 on luciferase intensity of the *ctnna2* enhancer region sequence (WT, rs12472215 A) as compared to a mutated sequence (A>T mutation). In D, F, and H, *p < 0.05, **p < 0.01 vs. Sham. In I and K, *p < 0.05, **p < 0.01. Two‐tailed unpaired t‐test was used in E, G, H, and I. Mann–Whitney U test was used in F. One‐way ANOVA was used in J and K, and Two‐way ANOVA was used in L. T + I: TNF‐α + isoflurane.

We have previously identified 6 SNP mutations on *CTNNA2* to be associated with increased susceptibility for PND in elderly patients undergoing cardiac surgery (*NCT02084030*) [10]. A bioinformatic research strategy was used to define which site is possibly related to *ctnna2* transcriptional alteration. In the Gene Set Enrichment Analysis (GSEA) data from Davis et al. [26], chr2:79425802 (GRCh38.p14, gene position of rs12472215 in the human genome) revealed high affinity to H3K4me1, which makes it a potential enhancer area (Figure 1A). To further test the hypothesis that this SNP mutation accounts for *CTNNA2* transcriptional change after surgery, we screened for alignment of TFs that bind to the 45 bp sequence centered on rs12472215 using JASPAR online tools (https://jaspar.elixir.no). Sequence alignment predicted 6 TFs that potentially bind to the sequence centered on rs12472215 (A) and 20 TFs that potentially bind to the sequence centered on rs12472215 (T) (Tables S2 and S3). The core binding sequence with GAAAAC or GTAAAC resulted in dramatically different predictive binding TFs, among which the mutated sequence T manifested significantly higher scores with several members of the Fox family (Figure 1B), making it a plausible target for transcriptional regulation. Sequences used for prediction were: ATCTACAGAGTATTGTATTCTGAAAACCTAACACACTAATATTGC and ATCTACAGAGTATTGTATTCTGTAAACCTAACACACTAATATTGC. Only predictions that contain the mutation sequence were preserved in the Supporting Information tables.

Among the first three predicted TFs that may bind and affect the transcription of *CTNNA2*, Foxo3 was reported to be neuroprotective [27] in neurodegenerative diseases like Parkinson's [28] and Huntington's disease [29]. However, there is also evidence that Foxo3 activation is associated with neuronal death [30], while phosphorylation of Foxo3 and its movement away from the nucleus would attenuate Alzheimer's disease (AD) – type amyloid neuropathology and preserve spatial reference memory [31]. The mixed evidence led to the possible involvement of Foxo3 in cognitive changes.

Using HADDOCK to mimic the spatial ligation of Foxo3 protein to a 10 bp region near the rs12472215 (A or T). Docking images showed closer interactivity of sequence T vs. A (Figure 1C). We therefore built an in vitro model to mimic surgery and anesthesia using T + I stimulation on human SH‐SY5Y neurons. Sequencing results showed that the rs12472215 site of SHSY5Y was the variant type T (Figure S2) sequence is shown in reverse complement. The stimulation significantly reduced *ctnna2* mRNA levels in SH‐SY5Y cells (Figure 1D).

To decipher whether Foxo3 affects the transcription start site (TSS) of *CTNNA2* and the status change under T + I in SH‐SY5Y cells, ChIP‐PCR was carried out. The results showed that H3K4me3 affinity to transcription start sites (TSS) was lower, but not significantly decreased after T + I exposure (Figure 1E, non‐parametric test). H3K4me1 affinity to the rs12472215 enhancer region (E1‐rs12472215) was higher and significantly reduced after T + I exposure (Figure 1F). H3K4me1 is an enzyme that facilitates promoter‐enhancer interaction and gene activation [32]. The reduction of affinity after T + I exposure indicates reduced transcriptive activation. The affinity of H3K27ac was not affected by T + I stimulation at TSS or ES‐rs12472215 region (Figure 1G). Foxo3's affinity to TSS was low, but its affinity to the ES‐rs12472215 region was high. T + I stimulation significantly enhanced Foxo3's affinity to ES‐rs12472215 (T), but not TSS (Figure 1H), indicating that Foxo3 may be involved in *CTNNA2* transcriptive repression at this new enhancer region in SH‐SY5Y cells. To further compare the effect of Foxo3 on the transcription of rs12472215 mutation subtypes, a reporter assay was conducted in H293T cells. Results showed that Foxo3 dose‐dependently affects the transcription of the rs1272215 enhancer sequence (Figure 1I). Another transcription factor in the Fox family, Foxo1, which is not enriched in neurons, does not affect the fluorescent signal of the rs12472215 enhancer sequence (Figure 1J). When comparing Foxo3's effect on the transcription of rs12472215 (A) to rs12472215 (T), results showed that the inhibitory effect of Foxo3 on transcription of rs12472215 (T) was stronger as compared to that of rs12472215 (A) (Figure 1K). The above results proved that Foxo3 acts as a transcriptional repressor of the predicted enhancer region near rs12472215 in the human *ctnna2* gene, with an exaggerated effect on the mutated sequence (T) compared to the WT (A) sequence.

### 3.2 Hippocampal α‐N‐Catenin Expression Is Reduced, With CA1 Neuron Dysfunction and Spine Formation Impairment in PND Mice

*Ctnna2‐* encoded α‐N‐catenin is only expressed in the nervous system [33]. Studies have shown that *ctnna2* reduction affects axon migration [34] and its overexpression is associated with excessive spinous spine growth [35]. However, it is unknown if *ctnna2* expression affects neuronal function in the hippocampus or cognitive ability in animals. It is also unknown if *ctnna2* expression is involved in PND in mice.

**FIGURE 2:**
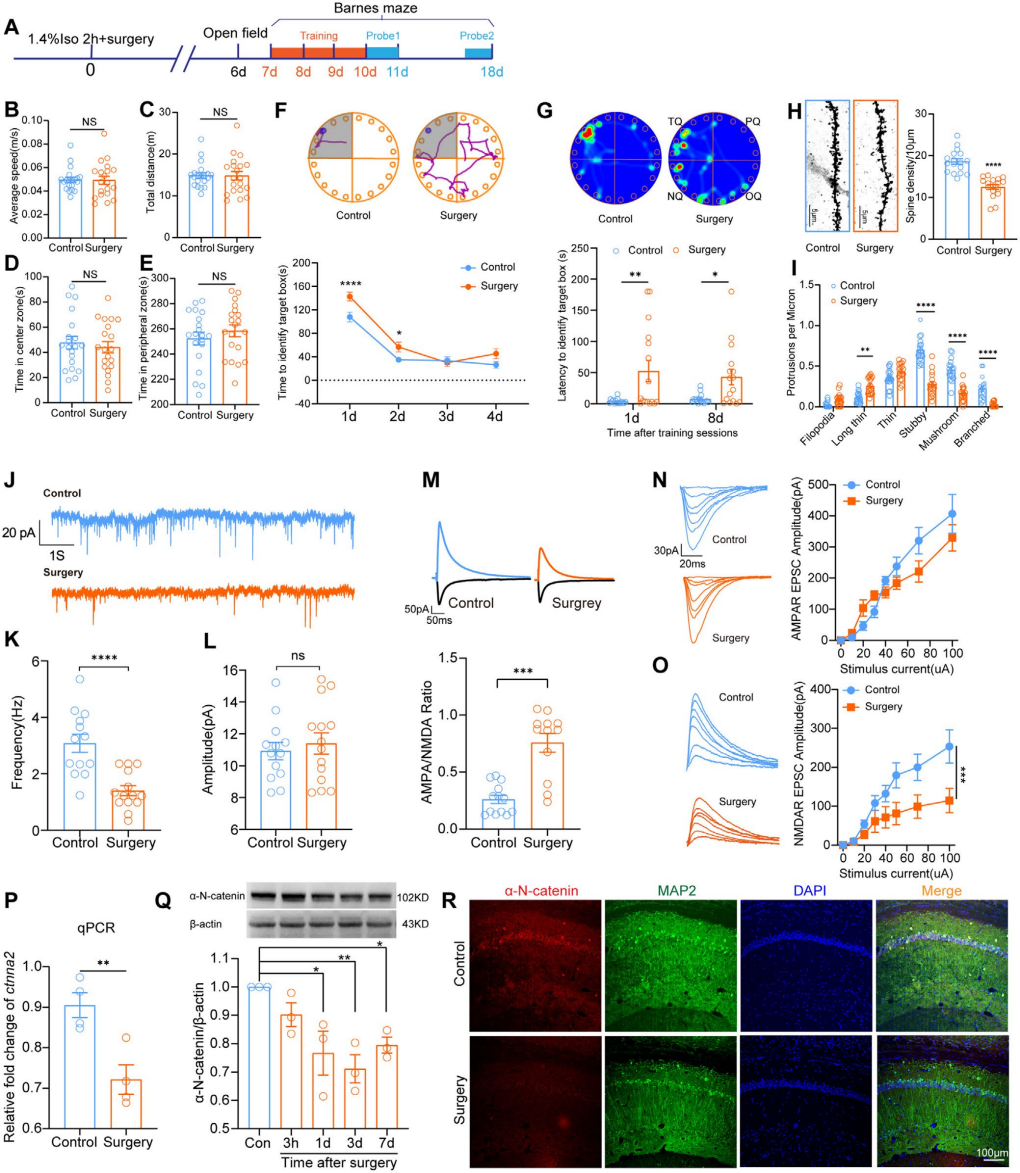
α‐N‐catenin reduction may be involved in the development of PND in mice. (A) Schematic illustration for the study protocol of behavioral tests. (B–E) Average speed, total distance traveled, time spent in the center zone, and time spent in the peripheral zone in the open field test. (F) Time to identify the target box during the learning phase in the Barnes maze test. Representative images showing the route of the mouse in each group. (G) Latencies to identify the target box during the test phase in the Barnes maze test at 1 day and 8 days after training. Representative image showing the heat map of mouse movement in each group. (H) Spine density of mice CA1 pyramidal neurons and representative Golgi staining image in each group. (I) Number of different categorized dendritic spines in the dendrites of CA1 neurons. (J) Representative images of spontaneous neuronal firing of CA1 pyramidal neurons in each group. (K) Statistical analysis of spontaneous firing frequency of CA1 pyramidal neurons. (L) Statistical analysis of spontaneous firing amplitudes of CA1 pyramidal neurons. (M) NMDA/AMPA amplitude ratio of CA1 neurons in different groups and representative current image. (N) AMPAR EPSC amplitude of CA1 pyramidal neurons in the hippocampal slice from each group. (O) NMDAR EPSC amplitude of CA1 pyramidal neurons in the hippocampal slice from each group. *ctnna2* mRNA transcription level (P), α‐N‐catenin expression (Q), and immunofluorescent staining of α‐N‐catenin (R) in the hippocampus of mice in different groups. *p < 0.05, **p < 0.01, ***p < 0.001 and ****p < 0.0001. Two‐tailed unpaired t test was used in D, E, K, L, and P. Mann–Whitney U test was used in B, C, and H. One‐way ANOVA was used in Q, and Two‐way ANOVA was used in F, I, G, N, and O.

To test if α‐N‐catenin expression is changed in the PND mouse model, we established both in vivo and in vitro models of PND. The timeline for the in vivo experiment with mice and behavioral tests is shown in Figure 2A. The open field test revealed no apparent changes in moving ability or anxiety behavior in mice 6 days after surgery (Figure 2B–E). PND model confirmed that mice suffered from learning and memory deficits after isoflurane anesthesia and surgery. The time to identify the target hole during training and the latency to identify the target hole at test phases (both 1d and 8 days after training) were both longer in mice in the surgery group compared to mice in the control group (Figure 2F,G). Golgi staining of the CA1 tissue revealed that the number of long and thin spines (unstable) was increased, and the number of stubby, mushroom, and branched spines (stable and matured ones) was decreased after surgery (Figure 2H,I). To further explore the functional changes of CA1 pyramidal neurons after surgery, ex vivo electrophysiological research showed that the spontaneous spiking frequency was reduced in CA1 pyramidal neurons after surgery, while the amplitude of EPSCs remained unchanged (Figure 2J–L). AMPAR/NMDAR EPSC amplitude ratio was reduced (Figure 2M), of which AMPAR EPSC amplitudes were similar (Figure 2N), but AMPAR EPSC amplitudes were decreased in CA1 neurons of mice after surgery. (Figure 2O).

To further explore the possible role of *ctnna2* transcription in the development of PND in mice, mRNA and protein levels were tested in the mouse hippocampus. Results showed that *ctnna2* transcription was significantly reduced at 3 days after anesthesia and surgery (Figure 2P). α‐N‐catenin protein content was also decreased after anesthesia and surgery, which remained decreased for at least 7 days after surgery (Figure 2Q). Immunofluorescent staining also showed that α‐N‐catenin was significantly lower in the CA1 neurons of mice after surgery (Figure 2R). These results proved that anesthesia and surgery affected neuronal morphology, electrical features, and α‐N‐catenin expression in the CA1 region. α‐N‐catenin might be involved in the development of spatial cognitive decline in mice after surgery.

### 3.3 Alteration of α‐N‐Catenin Expression in the Hippocampus Affected CA1 Pyramidal Neuron Function and Spatial Cognition in Mice

**FIGURE 3:**
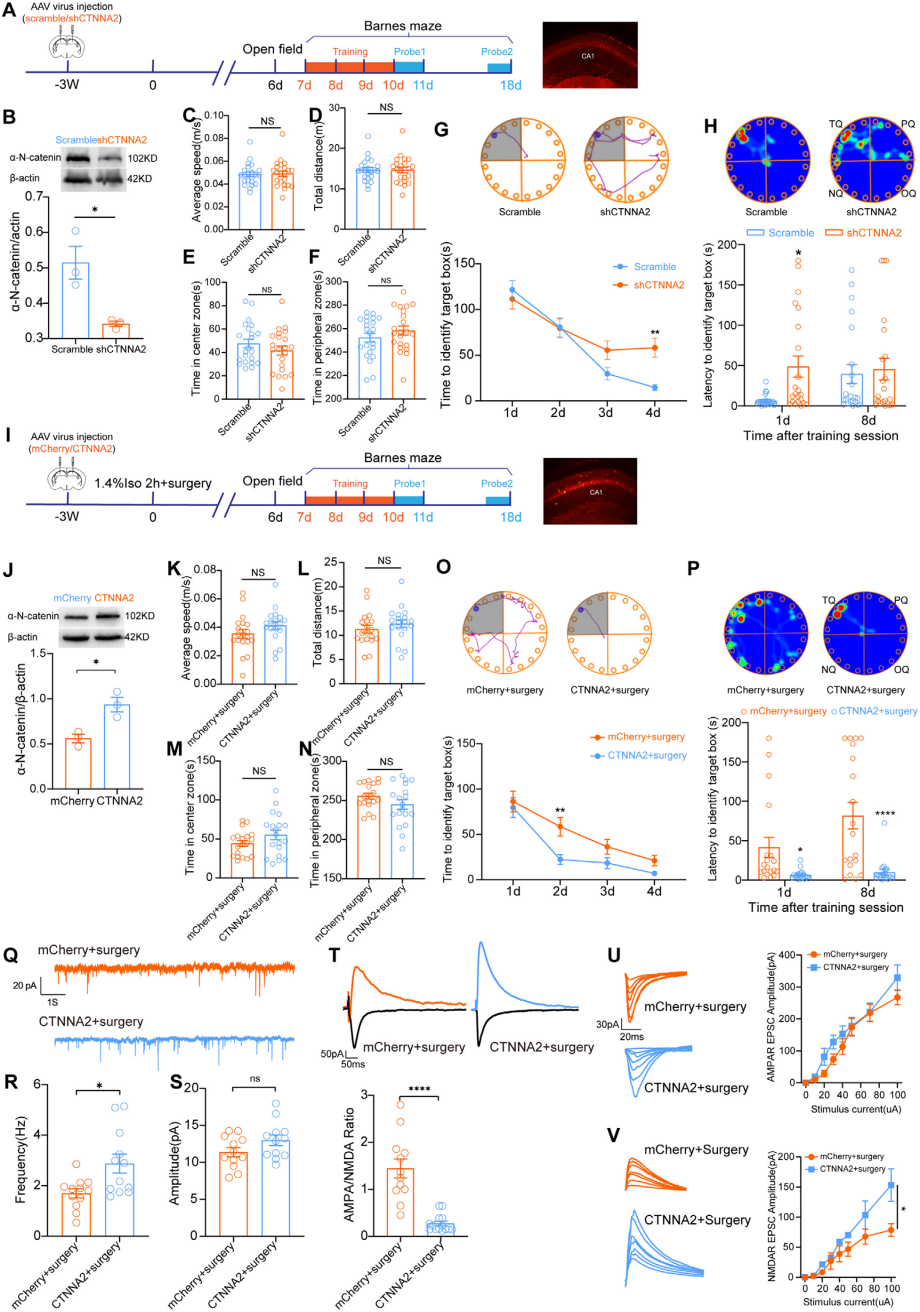
α‐N‐catenin reduction in the hippocampus induces spatial cognitive impairment in PND mice. (A) Schematic illustration for study protocol of rAAV injection and behavioral tests in mice receiving virus containing a scramble or shCTNNA2 sequence. Representative image showing that the virus containing mCherry infected CA1 neuron of the hippocampus. (B) α‐N‐catenin expression in the hippocampus after rAAV injection. (C– F) Average speed, total distance traveled, time spent in the center zone, and time spent in the peripheral zone in the open field test. (G) Time to identify the target box during the learning phase in the Barnes maze test. Representative images showing the route of the mouse in each group. (H) Latencies to identify the target box during the test phase in the Barnes maze test at 1 day and 8 days after training. Representative image showing the heat map of mouse movement in each group. (I) Schematic illustration for study protocol of rAAV injection and behavioral tests in mice receiving virus w/o *ctnna2* sequence. (J) α‐N‐catenin expression in the hippocampus after rAAV injection. (K–N) Average speed, total distance traveled, time spent in the center zone, and time spent in the peripheral zone in the open field test of mice from each group. (O) Time to identify the target box during the learning phase in the Barnes maze test. Representative images showing the route of the mouse in each group. (P) Latencies to identify the target box during the test phase in the Barnes maze test at 1 day and 8 days after training. Representative image showing the heat map of mouse movement in each group. (Q) Representative images of spontaneous neuronal firing of CA1 pyramidal neurons in each group. (R) Statistical Analysis of spontaneous firing frequency of CA1 pyramidal neurons. (S) Statistical Analysis of spontaneous firing amplitudes of CA1 pyramidal neurons. (T) NMDA/AMPA amplitude ratio of CA1 neurons in different groups and representative current image. (U) AMPAR EPSC amplitude of CA1 pyramidal neurons in the hippocampal slice from each group. (V) NMDAR EPSC amplitude of CA1 pyramidal neurons in the hippocampal slice from each group. *p < 0.05, **p < 0.01 and ****p < 0.0001. Two‐tailed unpaired *t* test was used in B–F, J–N, and S. Mann–Whitney *U* test was used in R and T. Two‐way ANOVA was used in G, H, O, P, U, and V.

To investigate whether α‐N‐catenin expression reduction is involved in cognitive decline in mice PND model, we conducted genetic manipulation to regulate α‐N‐catenin expression with AAV. The study protocol is shown in Figure 3A. Western blot analysis of the hippocampus 3 weeks after infection showed that rAAV‐hSyn‐shCTNNA2‐P2A‐mCherry‐WPREs virus, which carried a short hairpin RNA of *ctnna2*, reduced α‐N‐catenin expression in the hippocampus of mice (Figure 3B). Open field test showed that travel speed, distance, time spent in the center zone, and time in the peripheral zone were similar between the two groups (Figure 3C–F). Downregulation of *ctnna2* induced spatial learning and short‐term memory defects in mice, revealed by the Barnes maze test (Figure 3G,H). On the other hand, upregulation of *ctnna2* was achieved by injection of rAAV‐hSyn‐CTNNA2‐P2A‐mCherry‐WPREs into the CA1 region in mice. The protocol and the effectiveness of *ctnna2* upregulation are shown in Figure 3I,J. Open field revealed no obvious changes (Figure 3K–N). These results indicated that moving ability and anxiety level were not altered by *ctnna2* overexpression in mice after anesthesia + surgery. Upregulation of *ctnna2* in CA1 neurons significantly improved spatial cognitive learning in mice that underwent anesthesia and surgery (Figure 3O). Spatial memory was also improved at 1 day and 8 days after training (Figure 3P). ex vivo electrophysiological recordings revealed that upregulation of *ctnna2* before surgery improved CA1 pyramidal neuron spontaneous spiking frequency, while their amplitude was unchanged (Figure 3Q–S). Specifically, the AMPAR/NMDAR ratio was reduced (Figure 3T). AMPAR EPSC amplitudes were unchanged, while NMDAR EPSC amplitudes were increased by *ctnna2* overexpression after surgery (Figure 3U,V).

### 3.4 Foxo3 Act as a Transcriptional Repressor for *ctnna2* in Mice, Partially by Inhibiting Ascl1‐Mediated Transcriptional Activation Through a Chromatin‐Interacting Manner

**FIGURE 4:**
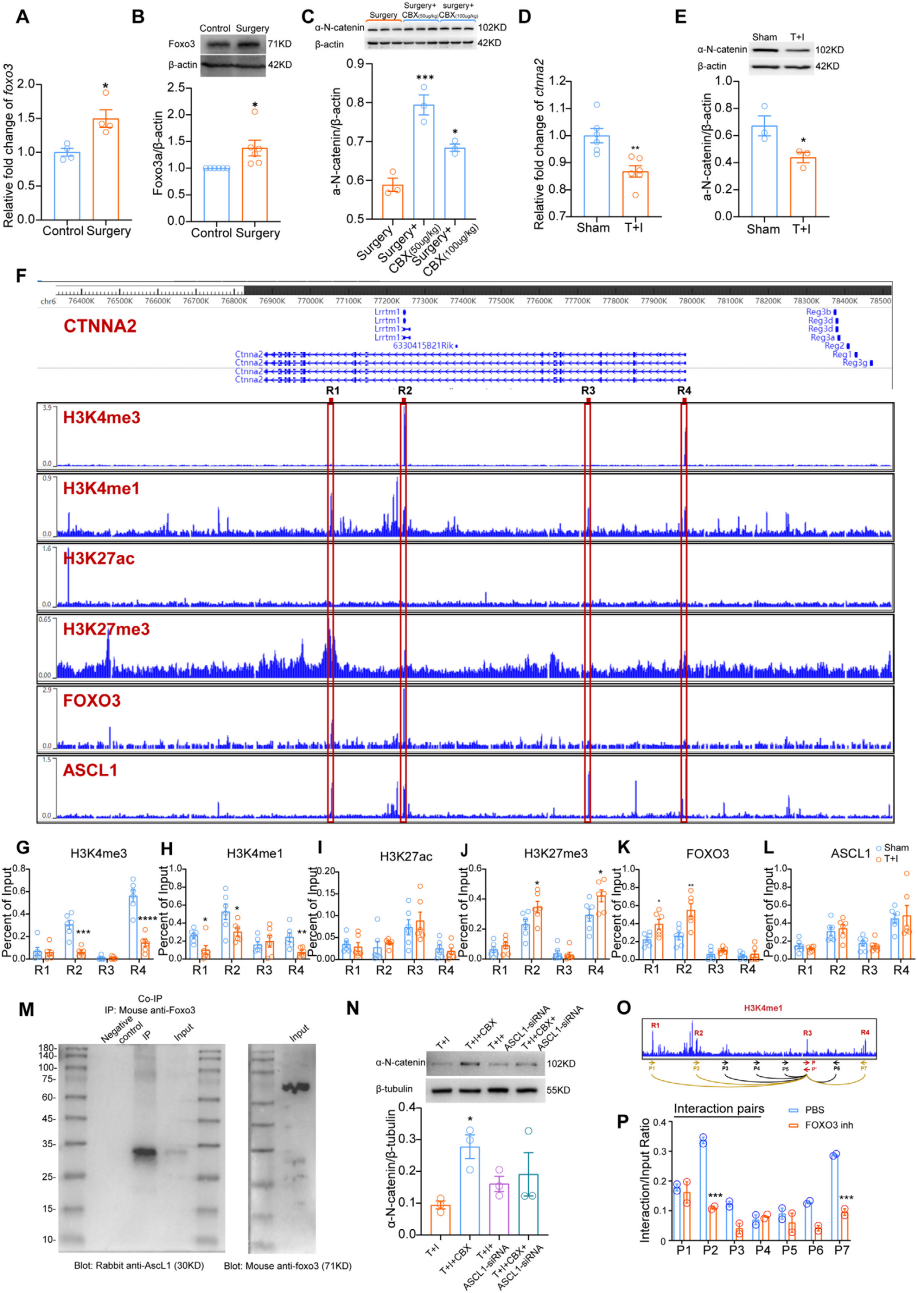
Foxo3/Ascl1‐regulated *ctnna2* transcriptional inhibition is involved in α‐N‐catenin reduction in mice. *Foxo3* mRNA level (A) and protein level (B) in the hippocampus of mice in different groups. *p < 0.05 vs. control. (C) Effect of Foxo3‐specific inhibitor (CBX) on α‐N‐catnein expression after surgery. *p < 0.05, ***p < 0.001 vs. surgery. α‐N‐catnein transcriptional level (D) and protein level (E) in primary cultured neurons w/o TNF‐α + iso stimulation. *p < 0.05, **p < 0.01 vs. sham. (F) DNA binding affinity of H3K4me3, H3K4me1, H3K27ac, H3K27me3, Foxo3, and Ascl1 to the mouse sequence of *ctnna2*. The four red quadrants, R1‐R4, represent hypothetical transcriptively active sequences that are tested for affinity by ChIP‐PCR. Data from Cistrome.org. Davis et al. and Webb et al. [26, 36]. (G–L) DNA binding Affinity of H3K4me3 (G), H3K4me1 (H), H3K27ac (I), H3K27me3 (J), Foxo3 (K), and Ascl1 (L) on four predicted transcriptionally active areas (R1‐R4) in primary cultured mouse cortical neurons w/o T + I stimulation. *p < 0.05, **p < 0.01, ***p < 0.001 and ****p < 0.0001 vs. Sham. (M) Co‐IP of mouse hippocampus tissue after surgery showing that Ascl1 physically interacts with Foxo3 in vivo. (N) α‐N‐catnein expression in neurons with different treatments. ****p < 0.0001 vs. sham. ^#^ *p* < 0.05 vs. T + I. (O) Illustrative image of interactive pairs compared with 3C experiment among R1‐R4. *p < 0.05 vs. T + I. (P) The effect of a universal Fox inhibitor on the interactive intensity of chromosome position pairs. ***p < 0.001 vs. PBS group. Two tailed unpaired t test was used in A, B, D, E, G (R2 and R4), H (R2‐R4), I (R3 and R4), J (R1, R2 and R4), K and L (R1‐R3) and P. Mann–Whitney U test was used in G (R1 and R3), H (R1), I (R1 and R2), J (R3) and L (R4). One‐way ANOVA was used in C, Kruskal‐Wallis test was used in N, and Two‐way ANOVA was used in P. T + I: TNF‐α + isoflurane.

As previously shown, Foxo3 dose dependently affects the transcription of the enhancer region around rs12472215 in the human *ctnna2* gene. However, this enhancer region is located within the 2nd intron. Although this region is highly conserved in primates and monkeys, it is not conserved in rodents like the mouse (Figure S3). No other report has indicated that Foxo3 regulates the expression of *ctnna2* in rodents. But the sequence that was predicted on JASPAR for Foxo3 transcriptional regulation (GTAAACCT) can be found in several parts of the *ctnna2* genome in mice. To further test the possible regulation of *cnnta2* by Foxo3 in mice and the underlying mechanism, we first explored the expression of Foxo3 in the mouse model of PND. Results showed that both the mRNA and protein content of Foxo3 were upregulated in the hippocampus of mice after surgery (Figure 4A,B). A specific inhibitor of Foxo3, CBX [37], was used to test if Foxo3 elevation in the hippocampus affected α‐N‐catenin expression. Intraventricular injection of both 50 μg kg^−1^ and 100 μg kg^−1^ CBX effectively enhanced α‐N‐catenin expression in the hippocampus in mice after surgery (Figure 4C), indicating that Foxo3 elevation may be involved in α‐N‐catenin repression.

To explore the transcriptional effective site of Foxo3 on *ctnna2* in mice, we first rebuilt the in vitro model of surgery and anesthesia in primary cultured embryonic cortical neurons of mice. A similar T + I protocol was used to mimic anaestheisa+surgery in vitro. T + I significantly inhibited both *ctnna2* transcription and protein expression in the cultured neurons (Figure 4D,E). Using Cistrome data viewer combined with ChIP‐PCR data, we confirmed 4 possible transcriptional regulatory regions (R1‐R4) in the *ctnna2* genome of mouse (Figure 3F). ChIP‐PCR comparison was conducted to identify sites that are transcriptionally active or inactive in sham or T + I conditions. The results revealed that H3K4me3 and H3K4me1 affinity to R2 and R4 sites were high at sham but reduced by T + I (Figure 4G,H). H3K4me1 affinity was also high to the R1 region, which was reduced by T + I (Figure 4H). But H3K27ac affinity to all 4 sites was unchanged after stimulation (Figure 4I). The affinity of H3K27me3 to the R1 and R2 regions was significantly increased after T + I (Figure 4J). These changes were in corroborate with Foxo3 affinity (Figure 4K). Since H3K27me3 is a functional gene repressor, these results indicate that Foxo3 may be actively involved in the repression of *cnnta2* transcription in mouse at R1 and R2. Foxo3 was reported to inhibit gene transcription through interactional inhibition of Ascl1's transcriptional activatory effect [36]. We also tested the affinity change of Ascl1 to R1‐R4. Results showed that Ascl1 can bind to all four sites, with stronger affinity to R3 and R4, but unchanged after T + I stimulation (Figure 4L). Co‐immunoprecipitation (Co‐IP) of the hippocampus extracts from mouse after surgery confirmed Foxo3‐Ascl1 interaction in vivo (Figure 4M), which further indicated that Foxo3 may elicit transcriptional repression effect by deactivating Ascl1. To confirm the transcriptional effect of Foxo3 on the *ctnna2* gene and its relationship with Ascl1, we conducted a western blot of α‐N‐catenin in neurons with or without Ascl1 siRNA and CBX treatment in the in vitro PND model. Results showed that CBX elevated α‐N‐catenin expression in T + I stimulated cells (Figure 4N). Ascl1 siRNA abolished the effect of CBX, indicating that Foxo3 elicits transcriptional inhibition of *ctnna2* by deactivating Ascl1(Figure 4N).

Although Ascl1 has a higher affinity at R3 and R4, this affinity was not altered by T + I stimulation. On the other hand, in R1 and R2, where Foxo3 affinity was higher and enhanced by T + I, Ascl1 affinity was lower. We speculated that spatial chromosome interaction may be affected by Foxo3. Therefore, a 3C experiment is carried out to test if the interaction pairs exist and are affected by the function of Fox. Seven interaction pairs were observed (Figure 4O). Results indicated that the interactions of P2, P6, and P7 were inhibited by Fox inhibitor (Figure 4P). These results indicated that R3 is closely interacting with R2 and R4, but this interaction depends on the function of Foxo3. Foxo3 elevation may inhibit *ctnna2* transcription at all four sites through inhibiting Ascl1's transcriptional activity by affecting chromosome spatial structure and accessibility.

### 3.5 Foxo3 Expression and Post‐Translational Acetylation Is Regulated by Sirt1 in the In Vitro Model of PND in Primary Cultured Mice Neurons

**FIGURE 5:**
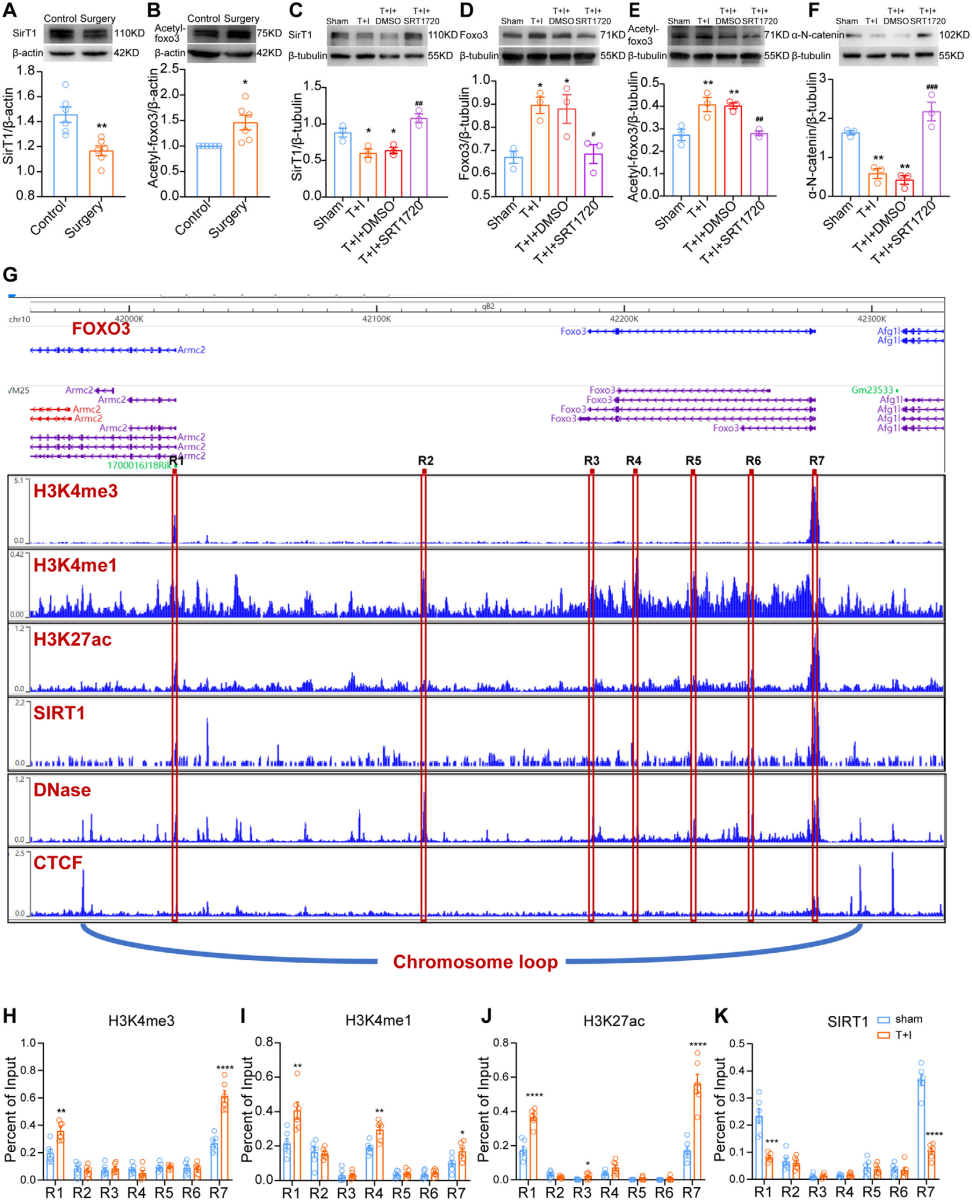
Sirt1 reduction after anesthesia and surgery regulates Foxo3 acetylation and *Foxo3* transcription. Sirt1 expression (A) and Acetyl‐Foxo3 level (B) in the hippocampus of mice from different groups. In vitro expression of Sirt1 (C), Foxo3 (D), Acetyl‐Foxo3 (E), and α‐N‐catenin (F) in the cultured primary cortical neurons. (G) DNA binding affinity of H3K4me3, H3K4me1, H3K27ac, and Sirt1 to the mouse sequence of *foxo3*. The red squares R1‐R7 represent hypothetical transcriptomic active sequences that are tested for affinity. Data from Cistrome.org. (H–K) DNA binding Affinity of H3K4me3 (H), H3K4me1 (I), H3K27ac (J) and Sirt1 (K), on seven predicted transcriptional active area (R1‐R7) in primary cultured mouse cortical neurons w/o T + I. *p < 0.05, **p < 0.01, ***p < 0.001 and ****p < 0.0001 vs. control or sham. ^#^ *p* < 0.05, ^##^ *p* < 0.01, ^###^ *p* < 0.001 vs. T + I group. Two tailed unpaired t test was used in A, B, G (R1, R2, R4‐R7), H, I (R1, R2, R4 and R7) and J. Mann–Whitney U test was used in G (R3), I (R3, R5 and R6) and J (R3). One‐way ANOVA was used in C, D, E, and F. T + I: TNF‐α + isoflurane.

It was reported that Foxo3 was mainly acetylated by Sirt1 and Sirt2 [38], of which Sirt1 was reported to decrease after surgery and anesthesia [39, 40]. Our western blot assay confirmed the reduction of Sirt1 expression in the hippocampus of mice after surgery (Figure 5A). Since Sirt1 mediates acetylation, acetyl‐Foxo3 levels were increased in the hippocampus of mice after surgery (Figure 5B).

To elucidate the role of Sirt1 on α‐N‐catenin expression, a Sirt1‐specific activator, SRT1270, was used in primary cultured mouse cortical neurons. SRT1270 significantly reversed the reduction of Sirt1 expression after T + I stimulation (Figure 5C), while down‐regulating Foxo3 expression (Figure 5D) and acetyl‐Foxo3 level (Figure 5E). α‐N‐catenin expression reduction was also reversed by SRT1270 after T + I stimulation (Figure 5F). The above results indicated that Sirt1 affects Foxo3 acetylation post‐translationally and that Sirt1 activation is an upstream regulator of α‐N‐catenin expression, possibly through regulation of Foxo3.

There was no evidence whether Sirt1 regulates *Foxo3* transcription. We also conducted ChIP‐PCR on 7 possible regulatory sites (Figure 5G, R1‐R7) on the gene of *foxo3* in mice. Site R1, although far from the *foxo3* gene, shares a CCCTC‐binding factor (CTCF) motif with *foxo3*. CTCF is a binding motif that bridges genome topology and function [41]. Genes within a CTCF binding motif always exhibit patterns in co‐expression. They may form a co‐transcriptional relationship that also regulates the transcription of *foxo3*. ChIP‐PCR results showed that the affinity of H3K4me3, H3K4me1, and H3K27ac to R1 and R7 is all upregulated after T + I stimulation (Figure 5H–J). At the same time, Sirt1's high affinity to R1 and R7 was reduced (Figure 5K). There is also an open frame at R4 where DNase affinity was high. ChIP‐PCR revealed that H3K4me1 affinity to R4 was increased after T + I (Figure 5I). These results indicated that R1, R4, and R7 may all be regulatory sites for *foxo3* transcription, of which R1 and R7 may be affected by Sirt1. Since Foxo3 expression was upregulated after T + I, Sirt1 may act as a transcriptional inhibitor of *foxo3* in mice. Reduction of Sirt1 after surgery and anesthesia may be the upstream mechanism of overexpressed Foxo3 and acetyl‐Foxo3 (more stable and resistant to ubiquitin‐mediated degradation) that caused *ctnna2* repression.

### 3.6 Interference of Foxo3‐*ctnna2* Interaction by a Decoy Oligodeoxynucleotide Reversed Postoperative α‐N‐Catenin Reduction and Prevented Cognitive Dysfunction After Surgery

As shown above, Foxo3 inhibits *CTNNA2* transcription in both the human SH‐SY5Y cell line and *ctnna2* transcription in the mouse PND model. The mutated rs12472215 (T) sequence may act as a possible decoy to block the interaction of Foxo3 on *ctnna2* perioperatively. Next, we aimed to design and test whether a proper decoy may prevent α‐N‐catenin reduction and reverse cognitive decline in the PND mouse model.

#### 3.6.1 Design of Decoy Oligodeoxynucleotides

**FIGURE 6:**
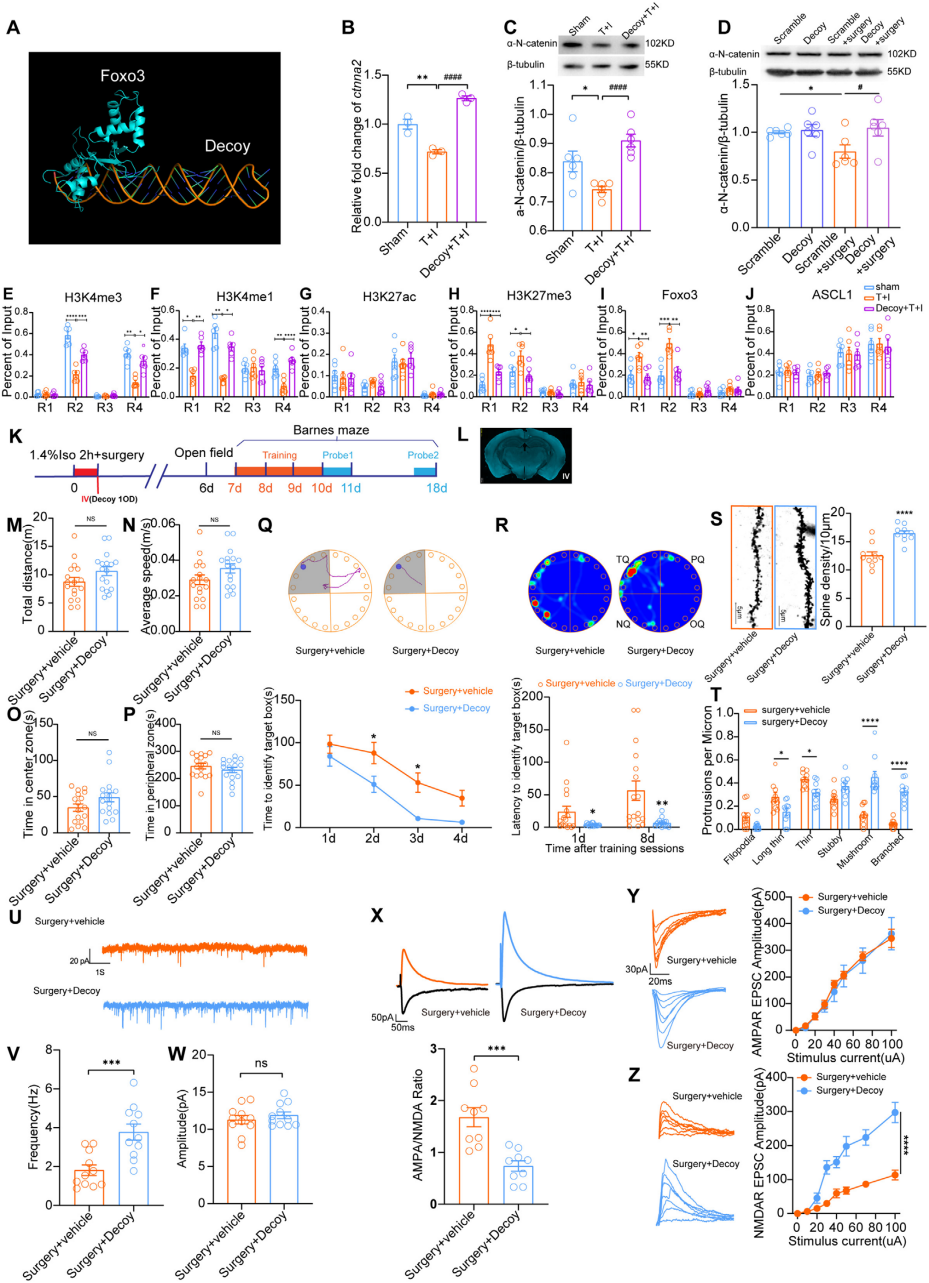
A newly synthesized decoy oligodeoxynucleotide (decoy) prevented *ctnna2* transcriptional repression and rescued mice from PND. (A) Representative image of Foxo3 interacting with the decoy sequence, illustrated with PyMol (Schrodinger, New York, NY). *ctnna2* mRNA level (B) and α‐N‐catnein protein level (C) in differentially treated primary cultured mice cortical neurons. (D) α‐N‐catnein expression in the hippocampus of mice treated with decoy or its scramble control in different groups. (E–J) DNA binding Affinity of H3K4me3 (E), H3K4me1 (F), H3K27ac (G), H3K27me3 (H), Foxo3 (I), and Ascl1 (J) on four predicted transcriptionally active areas (R1‐R4) in primary cultured mouse cortical neurons w/o T + I stimulation and/or decoy treatment. (K) Schematic illustration for study protocol of decoy or vehicle injection and behavioral tests in mice. (L) Representative fluorescent image of mouse brain slice showing FITC‐tagged decoy deposition in neurons at 2 h after intravenous injection. (M‐P) Average speed, total distance traveled, time spent in the center zone, and time spent in the peripheral zone in the open field test. (Q) Time to identify the target box during the learning phase in the Barnes maze test. Representative images showing the route of the mouse in each group. (R) Latencies to identify the target box during the test phase in the Barnes maze test at 1 day and 8 days after training. Representative image showing the heat map of mouse movement in each group. (S) Spine density of mice CA1 pyramidal neurons and representative Golgi staining image in each group. (T) Number of different categorized dendritic spines in the dendrites of CA1 neurons. (U) Representative images of spontaneous neuronal firing of CA1 pyramidal neurons in each group. (V) Statistical Analysis of spontaneous firing frequency of CA1 pyramidal neurons. (W) Statistical Analysis of spontaneous firing amplitudes of CA1 pyramidal neurons. (X) NMDA/AMPA ratio of CA1 neurons in different groups and representative current image. (Y) AMPAR EPSC amplitude of CA1 pyramidal neurons in the hippocampal slice from each group. (Z) NMDAR EPSC amplitude of CA1 pyramidal neurons in the hippocampal slice from each group. In B, C, D, E, F, H, and I, *p < 0.05, **p < 0.01, ***p < 0.001, ****p < 0.0001, ^#^ *p* < 0.05, and ^####^ *p* < 0.0001. In Q‐Z, *p < 0.05, **p < 0.01, ***p < 0.001 and ****p < 0.0001 vs. surgery+vehicle group. A tailed unpaired t‐test was used in B, C, D, M‐P, S, and V‐X. Mann–Whitney U test was used in G (R1 and R3), H (R1), I (R1 and R2), J (R3), and L (R4). One‐way ANOVA was used in E (R1 and R2), F (R3 and R4), G (R2 an R3), H, I (R1‐R3), and J. C, Kruskal‐Wallis test was used in E (R3 and R4), F (R1 and R2), G (R1 and R4) and I (R4) and Two‐way ANOVA was used in Q, R, T, Y and Z. T + I: TNFα+isoflurane.

To disrupt the transcriptional repressive effect of Foxo3 on *ctnna2*, we designed a decoy oligodeoxynucleotide drug specifically based on the mutated rs12472215 sequence. The decoy sequence is shown below: 5′‐tgTTCTGtAAACCctagTTCTGtAAACCtattttttataGGTTTaCAGAActagGGTTTaCAGAAc. The hypothetical interaction between Foxo3 protein and the decoy created by PyMOL (Schrödinger, New York, NY) is shown in Figure 6A.

#### 3.6.2 Decoy Affect *ctnna2* Transcription Both in vivo and in vitro

To confirm if the decoy prevents Foxo3‐induced *ctnna2* transcriptional repression, in vitro and in vivo models were built. in vitro experiments showed that 0.2 OD decoy delivery before T + I stimulation significantly reversed the reduction of *ctnna2* transcription (Figure 6B) and α‐N‐catenin expression (Figure 6C) in primary cultured neurons. Intravenous injection of 1 OD decoy once before surgery significantly increased hippocampus α‐N‐catenin expression at 3 days after surgery, but its scrambled control decoy did not manifest any effect (Figure 6D).

#### 3.6.3 The Decoys Inhibit TNF‐α + Isoflurane Induced *ctnna2* DNA Affinity Change of H4K4me3, H3K4me1, Foxo3, and H3K27me3 Binding to *ctnna2*

ChIP‐PCR results showed that 0.2 OD decoy administered to the primary neuronal culture of mice reversed H3K4me3 affinity reduction at R2 and R4 caused by T + I stimulation (Figure 6E). Similarly, the reductions of H3K4me1 affinity to R1, R2, and R4 induced by T + I were reversed by the decoy (Figure 6F). H3K27ac affinity to all four sites on *ctnna2* remained similar after decoy treatment. Also, H3K27me3 (Figure 6G) affinity elevation caused by T + I stimulation at R1 and R2 was both blocked by decoy treatment (Figure 6H). Most importantly, Foxo3 affinity elevations at the R1 and R2 sites caused by T + I were both blocked by the decoy (Figure 6I), showing that this decoy exerted inhibition of Foxo3 binding on the predicted enhancer sites of the *ctnna2* gene in mice. As predicted, Ascl1 affinity to *ctnna2* at these sites was not affected by the decoy (Figure 6J).

#### 3.6.4 DecoyODNs Prevented Postoperative Neurocognitive Decline, Inhibited Dendritic Malformation, and Electrophysiological Dysfunction of CA1 Neurons in Mice After Anesthesia and Surgery

Schematic plan of behavioral test is shown in Figure 6K. fluorescein isothiocyanate (FITC)‐tagged decoy injected intravenously penetrated the blood‐brain barrier and showed fluorescence in the whole mouse brain at 2 h after medication (Figure 6L). The results showed that the decoy did not affect open field performance in mice (Figure 6L–O). But the decoy treatment significantly improved spatial learning (Figure 6M) and memory (Figure 6 N) in mice after surgery. Morphological evaluation of Golgi staining showed that dendritic spine density was increased (Figure 6O). The number of long, thin spines and thin spines are decreased, but the number of stubby, mushroom, and branched spines is increased in decoy‐treated mice after surgery (Figure 6P). ex vivo electrophysiological tests showed that spontaneous EPSC frequency is increased, but the spontaneous EPSC amplitude remained unchanged after decoy treatment (Figure 6Q–S). AMPAR/NMDAR ratio is reduced in decoy‐treated mice CA1 neurons (Figure 6T). NMDAR EPSC amplitude is increased in decoy‐treated neurons after surgery (Figure 6U), and AMPAR EPSC amplitude remains similar between groups (Figure 6V). These results together showed that decoy elicits a neuroprotective effect in mice undergoing surgery and anesthesia by blocking the transcriptive repressive effect of Foxo3 on *ctnna2*, and reversed the synaptic morphological deficit and synaptic transmissive dysfunction, especially NMDAR EPSC amplitude in CA1 neurons.

## 4 Discussion

Here, we present novel data revealing the transcriptional regulatory mechanism of Foxo3 on *CTNNA2* at a newly defined enhanced region near rs12472215 within an intron in the human *CTNNA2* gene. We showed for the first time that α‐N‐catenin reduction serves as a pathophysiological basis for PND. Changes in the expression of α‐N‐catenin in the hippocampus were shown to influence CA1 pyramidal neuron activity and cognitive function in mice. We also identified a new Sirt1‐foxo3‐*ctnna2* regulatory axis in neurons and the transcriptional regulatory sites within *foxo3* and *ctnna2* underlying the spatial cognitive dysfunction in PND mice. A decoy oligodeoxynucleotide was conjugated that prevents the reduction of α‐N‐catenin expression in neurons after surgery by disrupting transcriptional repression of Foxo3 on *ctnna2* in mice. More importantly, this decoy therapy not only prevented the morphological and functional deficits in CA1 neurons but also preserved cognitive function in the PND mice model.

### 4.1 The Effect of *ctnna2* Transcription in the Model of PND

The *Ctnna2* gene encodes neuronal alpha‐catenin (α‐N‐catenin) in both humans and rodents, with primary distribution in the nervous system. *Ctnna2* mRNA is expressed in neurons and astrocytes of the human brain and mouse models. Through its interaction with β‐catenin or plakoglobin, α‐N‐catenin indirectly anchors the transmembrane cell–cell adhesion molecule E‐cadherin to the actin cytoskeleton, initiating the binding activity of the actin filament [42]. It is also involved in the negative regulation of actin aggregation mediated by the Arp2/3 complex [43], which can affect the extension and retraction of pseudopodia and thus regulate the development of neuronal migration and neural projection [34]. α‐N‐catenin also plays a crucial role in cadherin‐mediated cell–cell adhesion and contributes to the establishment and stabilization of synapses [35]. The stabilization of synapses underlies the plasticity of neuronal spines and the formation of memory. However, its specific role in learning and memory function in adult animal models remains unexplored.

Takeichi et al. described how the absence or overexpression of α‐N‐catenin influences synaptic stability [11]. In the absence of α‐N‐catenin, synaptic contact is unstable, resulting in abnormal extension and contraction of the spinous head, excessive elongation of filamentous spines, but an inability to produce stable mushroom‐like spinous processes. Overexpression of α‐N‐catenin suppresses spine turnover, leading to the formation of redundant spines, which can result in large, deformed, or entangled spinous heads. These findings suggest that α‐N‐catenin homeostasis is crucial for synaptic plasticity, especially for the growth and maturation of the spinous process. But it remains unclear if α‐N‐catenin expression and neuronal spine morphology are altered after anesthesia and surgery, or how these changes would affect neuronal function. Using a mouse model of PND, we found that α‐N‐catenin mRNA level and protein content were decreased in the hippocampus after anesthesia and surgery, with the lowest protein expression at 3 days postoperatively (see Figure 2P,Q). This reduction correlated with decreased spine density, increased number of unstable spines, and decreased number of stubby, mushroom, or branched spines (see Figure 2H,I). CA1 neurons that expressed less α‐N‐catenin also exhibited electrophysiological defects, including reduced spontaneous frequency, decreased NMDAR EPSCs amplitude, whereas AMPAR‐mediated EPSCs amplitude remained unchanged (Figure 2J–O). These differences led to an increased AMPAR/NMDAR ratio after surgery. It is well established that NMDAR‐mediated postsynaptic transmission is highly plastic and is essential for the initiation of LTP [35]. Evidence also turns toward a balance that the location of NMDAR‐dependent LTP is postsynaptic [44]. In a study with an Alzheimer's disease mouse model, Tozzi et al. revealed that in 6‐month‐old mice, β‐amyloid plaques induced loss of LTP in CA1 that was linked to reduced NMDA/AMPA current ratio [45]. This is corroborated by our finding that the AMPA/NMDA current ratio was elevated in memory‐impaired mice after surgery (Figure 2M). Our results highlighted the role of impaired synaptic transmission mediated by NMDAR, but not AMPAR, in the development of PND.

To further clarify the relationship between α‐N‐catenin expression and cognitive function, we down‐regulated α‐N‐catenin in neurons in the hippocampal region with rAAV‐syn‐shCTNNA2‐mCherry virus and found that it replicates the spatial learning and short‐term memory impairment caused by anesthesia and surgery (see Figure 3A–H). Conversely, overexpression of α‐N‐catenin in the CA1 neurons via rAAV‐syn‐CTNNA2‐mCherry virus partially prevented spatial learning and memory deficits in mice after surgery (see Figure 3I–P). Moreover, α‐N‐catenin overexpression rescued electrophysiological changes caused by anesthesia and surgery in the CA1 pyramidal neurons. These results demonstrate that α‐N‐catenin expression in CA1 neurons directly influences spatial cognitive function in adult animals. However, the mechanism underlying the downregulation of α‐N‐catenin following anesthesia and surgery remains unclear.

### 4.2 Regulatory Mechanism of Foxo3 on *ctnna2* in PND

Of all six SNP mutations in *ctnna2* provided by GWAS screening, bioinformatic research indicated rs12472215 to be a possible functional target. JASPAR analysis predicted Fox family TFs binding to the 45 bp surrounding rs12472215 after the A>T mutation (Table S3). Q‐PCR and western blot analysis both revealed that Foxo3 is upregulated in the cultured neurons or hippocampus of mice after surgery and anesthesia stimulation (Figure 4A,B). Foxo3 may act as a transcriptional activator [46] or repressor [46, 47], depending on the target gene. However, no previous report has connected Foxo3 to *ctnna2* transcription. Reporter assay in human cell lines proved that Foxo3 inhibits transcription of the enhancer sequence near rs12472215 in a dose‐dependent pattern (Figure 1J). H3K4me1 affinity to this area is high and is reduced after T + I stimulation. The affinity of Foxo3 to the TSS is quite low, but to the E1‐rs12472215 is high, and the latter was significantly enhanced after T + I stimulation (Figure 1I). To assess the mutation's effect, Foxo3 repression on the mutated (T) versus WT (A) sequences was compared. Stronger repression was found on the mutated sequence than on the WT sequence (Figure 1L). Furthermore, ChIP‐PCR revealed that H3K4me1 has high affinity to the E1‐rs12472215 region, which can be regulated by T + I stimulation. And Foxo3's affinity to the E1‐rs12472215 region was also high and can be enhanced by T + I stimulation (Figure 1G,I). The above evidence indicates that rs12472215 is located at an enhancer region that can be regulated by Foxo3. Patients with rs12472215 (T) are more susceptible to Foxo3‐mediated transcriptional repression. However, this enhancer area is not conserved in rodents (Figure S3). It is unknown if *ctnna2* changes after anesthesia and surgery are also mediated by Foxo3 in mice.

Foxo3, often referred to as the “youth gene” [48], is the second‐highest differentially expressed gene in extreme longevity in humans. It is involved in multiple biological processes such as autophagy, DNA damage, antioxidant, cell cycle, and metabolism. Foxo3 knockout mice develop hearing loss due to synaptic localization defects in the ear [49]. In Parkinson's disease, inhibition of Foxo3 causes oxidative damage and is detrimental if the inhibition is too strong [50]. These results suggest a neurophysiological function and antioxidative role of Foxo3. However, Foxo3 has also been found to mediate acute axonal degeneration caused by the absence of neurotrophic factors [51], while consistent activation of Foxo3 may lead to acute apoptosis of dopaminergic neurons [50]. This evidence highlights the importance of Foxo3 homeostasis for normal nervous system function. But no previous studies have explored the role of Foxo3 in synaptic plasticity, learning, and memory function in the settings of any neural disorders, including PND. Therefore, we conducted chemical inhibition against Foxo3 to explore if Foxo3 would affect *ctnna2* expression in the settings of PND in mice. Indeed, intracerebral ventricular injection of a specific Foxo3 inhibitor CBX [37] effectively prevented α‐N‐catenin reduction in brain tissue of PND mice (Figure 4C), further corroborating the regulatory effect of Foxo3 on *ctnna2* transcription. ChIP‐PCR was performed on cultured mouse cortical neurons to identify Foxo3 target sites on the *ctnna2* sequence. Among four predicted regulatory regions (R1‐R4, Figure 4F), H3K4me3 binding was reduced in R2 and R4 areas (Figure 4G), H3K4me1 binding was reduced in R1, R2, and R4 areas (Figure 4H), while H3K27me3 binding was increased in R2 and R4 areas (Figure 4J). In alignment with the above changes, Foxo3 binding affinity significantly increased at R1 and R2 following T + I stimulation (Figure 4K). These results together showed that the increased Foxo3 elicits transcriptional repression on *ctnna2* in a neuronal model of PND at two newly defined enhancer sites ‐ R1 and R2. Since it was reported that Foxo3 often partners with Ascl1, which showed moderate to strong binding to all four sites but was not affected by T + I stimulation (ChIP‐PCR results), we conducted co‐IP in the hippocampal tissue of mice after surgery. The result indicated a strong binding of Foxo3 to Ascl1 (Figure 4M). But since Ascl1 binding to R3 and R4 sites is stronger than that of R1 and R2 sites, and Foxo3 only binds to R1 and R2, it is unknown how Foxo3 interactively affect *ctnna2* transcription through Ascl1. in vitro experiments using Ascl1 siRNA indicated that CBX elicited α‐N‐catenin preservation after T + I treatment was abolished by Ascl1 siRNA, indicating that Foxo3's transcriptional repression on *ctnna2* may be partially due to its repression of Ascl1's pro‐transcriptional effect (Figure 4N). 3C analysis revealed stronger R2‐R3 interaction and R4‐R3 interaction under normal conditions. But a universal Fox inhibitor blocked these interactions (Figure 4O,P). These findings suggest that Fox influences the *ctnna2* chromosome loop structure. The upregulation of Foxo3 after T + I stimulation may elicit a strong inhibitory effect on *ctnna2* transcription directly at R1 and R2, but also indirectly through Ascl1 by increased chromosome spatial interaction at R3 and R4.

### 4.3 Reduction of Sirt1 Regulates Foxo3 Acetylation and *foxo3* Transcription That Induced α‐N‐Catenin Reduction in PND

FoxO proteins undergo a variety of post‐translational modifications, such as phosphorylation, acetylation, ubiquitination, and arginine methylation [52]. Among them, the acetylation/deacetylation of Foxo3 protein dynamically regulates its biological function by affecting its DNA‐binding activity, stability, and interaction with other proteins [53]. The impact of Foxo3 acetylation on its transcriptional regulatory effect is controversial. Acetylation sites primarily reside in the DNA‐binding domain, and lysine acetylation has been shown to reduce their DNA‐binding capacity [54]. However, Foxo3 acetylation has also been shown to promote its transcriptional regulatory activity [55]. Acetylation of Foxo3 promotes cytosolic relocalization and ubiquitin‐mediated degradation [38, 56], while reducing acetylation retains Foxo3 in the nucleus, enhancing its transcriptional regulatory effect. Acetylated Foxo3 is mainly deacetylated by Sirt1 and/or Sirt2 in a nicotinamide adenine dinucleotide (NAD+) dependent manner. It was reported that after surgery, the expression of Sirt1 in the hippocampus is decreased [39, 40], potentially impairing Foxo3 deacetylation, increasing its acetylation level, thus reducing degradation, and amplifying transcriptional repression on *ctnna2*. We thereby verified Sirt1 expression drop in vivo in the hippocampus of PND mice (Figure 5A). Acetylated Foxo3 is increased after surgery and anesthesia in the mouse hippocampus (Figure 5B). in vitro experiments also revealed that Sirt1 is decreased and acetyl‐Foxo3 is increased after T + I stimulation. A specific Sirt1 activator, SRT1720, delivered before T + I stimulation, improved Sirt1 expression (Figure 5C) and reduced acetyl‐Foxo3 level (Figure 5D) in neurons after T + I. SRT1720 also restored α‐N‐catenin levels following T + I stimulation (Figure 5E). More importantly, ChIP‐PCR for *foxo3* gene in mice revealed that, at R1 and R7 sites, although H4K3me1, H3K4me3, and H3K27ac affinity increases, Sirt1 affinity is significantly decreased after T + I stimulation (Figure 5G–J). These results indicated that Sirt1 is also involved in *foxo3* transcription and may act as a transcription repressor on *foxo3*. Decreased affinity of Sirt1 to the R1 and R7 sites, possibly due to the reduced level of Sirt1, is also the two sites where histone methyltransferase and acetylase affinity were increased after T + I stimulation. Therefore, Sirt1 reduction after T + I may influence *ctnna2* transcription by modulating both Foxo3 expression and acetylation.

Together, the evidence highlights a Sirt1‐foxo3‐ctnna2 regulatory axis in the pathogenesis of PND in mice. After surgery and anesthesia, reduced Sirt1 levels impair Foxo3 deacetylation and transcriptional regulation. The resulting elevation of total Foxo3 and acetylated Foxo3 leads to transcriptional repression of *ctnna2*, ultimately causing CA1 neuron dysfunction and spatial cognitive deficit in mice.

### 4.4 Decoy ODNs and Their Effect

Hairpin‐type decoy oligodeoxynucleotides (ODNs) originate from the idea of antisense oligonucleotides (ASO) therapy, which treats a target RNA as a receptor for an ASO that forms a base pair and disturbs the transcriptional process [57]. Marco Cattaruzza et al. reported a 786C/T SNP in the human NOS‐3 gene associated with coronary heart disease, developed a decoy oligonucleotide, and proved that the decoy reduced shear stress‐induced NOS‐3 expression [58]. Here, we reported a previously unknown enhancer site of *ctnna2* and proved the transcriptive repressive effect of Foxo3 on *ctnna2* expression. We then designed a decoy that mimics the mutated rs12472215 sequence, which has a higher affinity to Foxo3. This decoy may interfere with the binding of Foxo3 to *ctnna2* DNA and disturb its transcriptive repressive effect. We then showed that a single dose of decoy could block α‐N‐catenin reduction both in vitro (0.5 OD) and for up to 3 days in vivo (1 OD) after surgery + anesthesia stimulation (Figure 6B–D). Since the DNA's single‐stranded nature of the hairpin makes it sensitive to single‐stranded endonucleases [59, 60], this decoy therapy puts less risk in altering the genome for a long time or affecting other biological functions of Foxo3. Furthermore, the decoy delivered to the culture medium before T + I stimulation blocked the increase of Foxo3's affinity to R1 and R2 sites in the mouse *ctnna2* gene revealed by ChIP‐PCR (Figure 6I). Interestingly, it also reversed the affinity change of H3K4me3, H3K4me1, and H3K27me3 on certain sites of *ctnna2* caused by T + I (Figure 6E,F,H), indicating that Foxo3 may also regulate the accessibility of the chromosome after T + I. The decoy, by inhibiting the Foxo3‐*ctnna2* interaction, also preserved the affinity of other histone lysine methylation enzymes to *ctnna2* in mice. These results indicate that this decoy effectively disturbed the binding of Foxo3 to *ctnna2* in mice. Most importantly, a single injection of decoy significantly prevented spatial learning and memory deficit in the mouse PND model (Figure 6K,P,Q). It also increased pyramidal neuron dendritic spine density, the number of matured spines, and prevented CA1 neuronal electrophysiological dysfunction after anesthesia and surgery (Figure 6R–Y). This decoy may elicit a neuroprotective effect in neurodegenerative diseases where Foxo3 elevation and *ctnna2* transcriptional inhibition are involved.

There are several limitations of this study. First, SNP screening results are from a clinical cohort of elderly patients who underwent cardiac surgery on cardiac bypass with propofol anesthesia. But our animal experiments are conducted in adult mice that underwent laparotomy with isoflurane anesthesia. It is not clear whether age and surgery type affect the expression of the Sirt1‐Foxo3*‐ctnna2* axis or if they are differentially regulated in elderly animals during the perioperative period. But the in vitro model uses inflammatory cytokine TNF‐α and isoflurane as stimulation, which may have a broader implication in diseases where neuroinflammation and volatile anesthesia are involved. Further studies are needed to explore if α‐N‐catenin is involved in human PND in other cohorts. Secondly, the pharmacokinetics and biosafety of the decoy oligodeoxynucleotides are not clear yet. It warrants further safety tests on animals and nonhuman primates to assess the possible benefit of oligonucleotide‐based therapy as a disease‐preventing therapy for PND and other potential neurodegenerative diseases that involve α‐N‐catenin reduction.

## 5 Conclusion

This study reveals the pivotal role of *ctnna2* transcriptional repression in the development of PND. It provided evidence that anesthesia and neuroinflammation induce Sirt1 reduction, enhance *Foxo3* transcription, and increase Foxo3 acetylation levels, which in turn bind to and repress *ctnna2* transcription of the mouse *ctnna2* gene that resulted in CA1 neuronal dysfunction and spatial memory deficit in mice. Foxo3 also binds to and represses *CTNNA2* in a human cell line at a newly defined enhancer region within the 2nd intron near rs12472215. The stronger repression of Foxo3 on rs12472215 (T) as compared to rs12472215 (A) may underlie the increased susceptibility of PND in rs12472215 (T) patients. More importantly, a decoy oligodeoxynucleotide that competitively binds to Foxo3 and disturbs its transcriptional repression effects on *ctnna2* is developed and proved to effectively prevent PND development in mice through rescuing α‐N‐catenin expression after anesthesia and surgery.

## Author Contributions

Conceptualization: Junlong Zhao and Jiao Deng. Data Curation: Zhixin Wu, Dongkun Xie, Jing Zhao, Jianshuai Zhao, Huiqing Liu, Dong Xing, Zhihong Lu, Tingting Gu, and Yaru Guo. Formal Analysis: Zhixin Wu, Dongkun Xie, Jing Zhao, Jianshuai Zhao, Huiqing Liu, Junlong Zhao, and Jiao Deng. Funding acquisition: Zhihong Lu, Hailong Dong, Junlong Zhao, and Jiao Deng. Investigation: Zhixin Wu, Dongkun Xie, Jing Zhao, Jianshuai Zhao, Huiqing Liu, Dong Xing, Tingting Gu, Yaru Guo, and Dan Wang. Methodology: Jiao Deng, and Junlong Zhao. Project administration: Jiao Deng and Junlong Zhao. Resources: Tingting Gu, Yaru Guo, and Dan Wang. Software: Jianshuai Zhao, Zhixin Wu, and Dan Wang. Supervision: Zhihong Lu, Hailong Dong, Junlong Zhao, and Jiao Deng. Validation: Jiao Deng and Junlong Zhao. Visualization: Zhixin Wu, Dongkun Xie, Jing Zhao, Jianshuai Zhao, and Huiqing Liu. Writing – original draft: Jiao Deng, Junlong Zhao, and Zhixin Wu. Writing – review and editing: Hailong Dong, Jiao Deng, and Jing Zhao.

## Ethics Statement

This work is approved by the Ethics Committee for Animal Experimentation of the Fourth Military Medical University on March 4th, 2023.

## Conflicts of Interest

All authors of the patent 202411787121.5 (application number) of the decoy and related reconstructed forms of this product mentioned in this manuscript are listed in this work. The rest of the authors declare no competing interests.

## Supporting information


Appendix S1.

Appendix S2.

Data S1.

## Data Availability

Data are deposited at https://data.mendeley.com/datasets/8j6dn4gdm8/1 and are ready to download. Availability of materials is listed in the method part.
