# Supplementary material for: A Decoy Oligodeoxynucleotides Disturbing Forkhead Box O3 Mediated ctnna2 Transcriptional Repression Prevents Postoperative Neurocognitive Disorder in Mice
Source: CNS Neurosci Ther. 2025 Aug 26;31(8):e70454. doi: 10.1111/cns.70454 (PMC12380581; doi:10.1111/cns.70454)
Supplement: Supplementary file 1 — Appendix S1. [file CNS-31-e70454-s003.docx]

**Supplementary Table 1. The sequences for qRT-PCR primers, Chip-qPCR and 3C.**

| **Gene Name** | **Primer sequences (5′-3′)** |
| --- | --- |
| CTNNA2 (Mus)-F | ACCAGTGGGAAAAGCAAGTCCG |
| CTNNA2 (Mus)-R | GTGTCCACATCTCCCTCTTGCA |
| FOXO3 (Mus)-F | CCTACTTCAAGGATAAGGGCGAC |
| FOXO3 (Mus)-R | GCCTTCATTCTGAACGCGCATG |
| ~~FOXO1 (Mus)-F~~ | ~~CTACGAGTGGATGGTGAAGAGC~~ |
| ~~FOXO1 (Mus)-R~~ | ~~CCAGTTCCTTCATTCTGCACTCG~~ |
| ACTB (beta Actin) (Mus)-F | CATTGCTGACAGGATGCAGAAGG |
| ACTB (beta Actin) (Mus)-R | TGCTGGAAGGTGGACAGTGAGG |
| rs12472215 ChIP (Human)1F: | 5’-CACAGCAATCAAGAATGC |
| rs12472215 ChIP (Human) 1R: | 5’-TACCCTTTTGATTAGAAGC |
| CTNNA2 TSS ChIP (Human)1F: | 5’-TACGGGCCACGGAAAGGT |
| CTNNA2 TSS ChIP (Human) 1R: | 5’-ACCCAGAGGCGGAGTGA |
| CTNNA2 ChIP (Mus) 1F | CTGCACACTTATCAGGAG |
| CTNNA2 ChIP (Mus) 1R | CAATCAGAGAGAAAGTCAT |
| CTNNA2 ChIP (Mus) 2F | ACCAGGACTGGTCTCCTC |
| CTNNA2 ChIP (Mus) 2R | TAAGACAGACTGTCCTGAA |
| CTNNA2 ChIP (Mus) 3F | TTCTTCACTACCAAGTAGAG |
| CTNNA2 ChIP (Mus) 3R | CATCCATATAACATAACTGA |
| CTNNA2 ChIP (Mus) 4F | AAGAGTCTGGCTGCTGAAG |
| CTNNA2 ChIP (Mus) 4R | TCAGCCCTTTGAGTATT |
| FOXO3 ChIP (Mus) 1F | TCAGCCTACAGATCCAGGA |
| FOXO3 ChIP (Mus) 1R  FOXO3 ChIP (Mus) 2F | TCCTCCTGGTCCCTGCTGC  ATAGCAGCCATAAGGAAGCA |
| FOXO3 ChIP (Mus) 2R | GCCATCTGCCCTCCTGA |
| FOXO3 ChIP (Mus) 3F | AGTATCTGAGGACGTTGGTA |
| FOXO3 ChIP (Mus) 3R | TAGGCACTCACAGAAAGTC |
| FOXO3 ChIP (Mus) 4F | CGTTGTCATGCAGACAGCCA |
| FOXO3 ChIP (Mus) 4R | CAATGTATATTATCACTCT |
| FOXO3 ChIP (Mus) 5F | AGGTGTGAACGTAGTGGATC |
| FOXO3 ChIP (Mus) 5R | CAGTGGGCTTTGTCATACTT |
| FOXO3 ChIP (Mus) 6F | GATCAGAGGAAAGAGCTT |
| FOXO3 ChIP (Mus) 6R | AATGTGACCAGATTGCTGT |
| FOXO3 ChIP (Mus) 7F | TTGGGGTCCCGCGCTCT |
| FOXO3 ChIP (Mus) 7R | TGTCAGGTTCTAATAAGGAA |
| 3C-P | TAGACTGACACATCAGAATA |
| 3C-P’ | AATCTCTCAGTATAGCAAC |
| 3C-P1 | AGGTCTATAATTACAGTCC |
| 3C-P2 | GTTGACTGAAACTGTAAG |
| 3C-P3 | CTGCACACTTATCAGGAGTT |
| 3C-P4 | TGGTCTCCTCTGCGCTCTGAT |
| 3C-P5 | TTCTTCACTACCAAGTAGAGTG |
| 3C-P6 | GAATATTCAGAGTTTAAGGT |
| 3C-P7 | CCCTATATCAGAGCTACATG |

**Supplementary Table 2. TFs that are predicted to bind with 45 bp sequence centered rs12472215 (A). Sequence used for prediction was:** ATCTACAGAGTATTGTATTCTGAAAACCTAACACACTAATATTGC

| **Matrix ID** | **Name** | **Score** | **Relative score** | **Start** | **End** | **Strand** | **Predicted sequence** |
| --- | --- | --- | --- | --- | --- | --- | --- |
| [MA0144.3](https://jaspar.elixir.no/matrix/MA0144.3) | MA0144.3.STAT3 | 8.76301 | 0.91572 | 18 | 26 | + | TTCTGAAAA |
| [MA1118.2](https://jaspar.elixir.no/matrix/MA1118.2) | MA1118.2.SIX1 | 7.63781 | 0.83232 | 23 | 31 | + | AAAACCTAA |
| [UN0597.1](https://jaspar.elixir.no/matrix/UN0597.1) | UN0597.1.ZNF182 | 6.92011 | 0.82229 | 23 | 34 | + | AAAACCTAACAC |
| [MA0137.4](https://jaspar.elixir.no/matrix/MA0137.4) | MA0137.4.STAT1 | 4.49186 | 0.83825 | 18 | 26 | + | TTCTGAAAA |
| [UN0610.2](https://jaspar.elixir.no/matrix/UN0610.2) | UN0610.2.ZNF337 | 4.34466 | 0.85186 | 19 | 26 | + | TCTGAAAA |
| [MA0766.3](https://jaspar.elixir.no/matrix/MA0766.3) | MA0766.3.GATA5 | 2.02595 | 0.82561 | 21 | 28 | + | TGAAAACC |

**Supplementary Table 3. TFs that are predicted to bind with the 45 bp sequence centered rs12472215 (T). Sequence used for prediction was:** ATCTACAGAGTATTGTATTCTGTAAACCTAACACACTAATATTGC

| **Matrix ID** | **Name** | **Score** | **Relative score** | **Start** | **End** | **Strand** | **Predicted sequence** |
| --- | --- | --- | --- | --- | --- | --- | --- |
| [MA0157.2](https://jaspar.elixir.no/matrix/MA0157.2) | MA0157.2.FOXO3 | 8.98405 | 0.88821 | 22 | 29 | + | GTAAACCT |
| [MA2118.1](https://jaspar.elixir.no/matrix/MA2118.1) | MA2118.1.FOXS1 | 8.8004 | 0.86529 | 21 | 28 | + | TGTAAACC |
| [MA0148.5](https://jaspar.elixir.no/matrix/MA0148.5) | MA0148.5.FOXA1 | 8.21718 | 0.86111 | 22 | 29 | + | GTAAACCT |
| [MA0031.2](https://jaspar.elixir.no/matrix/MA0031.2) | MA0031.2.FOXD1 | 7.69379 | 0.84954 | 22 | 28 | + | GTAAACC |
| [MA0481.4](https://jaspar.elixir.no/matrix/MA0481.4) | MA0481.4.FOXP1 | 7.66354 | 0.87337 | 22 | 28 | + | GTAAACC |
| [UN0802.1](https://jaspar.elixir.no/matrix/UN0802.1) | UN0802.1.FOXM1 | 7.64252 | 0.87024 | 22 | 28 | + | GTAAACC |
| [MA0852.3](https://jaspar.elixir.no/matrix/MA0852.3) | MA0852.3.FOXK1 | 7.53253 | 0.86678 | 22 | 28 | + | GTAAACC |
| [MA0047.4](https://jaspar.elixir.no/matrix/MA0047.4) | MA0047.4.FOXA2 | 7.45404 | 0.86678 | 21 | 28 | + | TGTAAACC |
| [MA2117.1](https://jaspar.elixir.no/matrix/MA2117.1) | MA2117.1.FOXP4 | 7.33817 | 0.85629 | 22 | 28 | + | GTAAACC |
| [MA1683.2](https://jaspar.elixir.no/matrix/MA1683.2) | MA1683.2.FOXA3 | 7.29276 | 0.85843 | 22 | 28 | + | GTAAACC |
| [MA1103.3](https://jaspar.elixir.no/matrix/MA1103.3) | MA1103.3.FOXK2 | 7.19895 | 0.85512 | 22 | 28 | + | GTAAACC |
| [MA0613.1](https://jaspar.elixir.no/matrix/MA0613.1) | MA0613.1.FOXG1 | 6.6492 | 0.8599 | 22 | 29 | + | GTAAACCT |
| [MA0033.2](https://jaspar.elixir.no/matrix/MA0033.2) | MA0033.2.FOXL1 | 6.20132 | 0.88894 | 22 | 28 | + | GTAAACC |
| [MA2098.1](https://jaspar.elixir.no/matrix/MA2098.1) | MA2098.1.ZNF766 | 6.1802 | 0.83642 | 21 | 29 | + | TGTAAACCT |
| [MA0850.1](https://jaspar.elixir.no/matrix/MA0850.1) | MA0850.1.FOXP3 | 5.53539 | 0.85495 | 22 | 28 | + | GTAAACC |
| [MA0848.1](https://jaspar.elixir.no/matrix/MA0848.1) | MA0848.1.FOXO4 | 5.19794 | 0.85142 | 22 | 28 | + | GTAAACC |
| [MA0849.1](https://jaspar.elixir.no/matrix/MA0849.1) | MA0849.1.FOXO6 | 4.91252 | 0.85714 | 22 | 28 | + | GTAAACC |
| [MA0846.2](https://jaspar.elixir.no/matrix/MA0846.2) | MA0846.2.FOXC2 | 4.33544 | 0.80636 | 19 | 29 | + | TCTGTAAACCT |
| [MA0593.2](https://jaspar.elixir.no/matrix/MA0593.2) | MA0593.2.FOXP2 | 4.20119 | 0.81 | 21 | 29 | + | TGTAAACCT |
| [MA0032.2](https://jaspar.elixir.no/matrix/MA0032.2) | MA0032.2.FOXC1 | 3.82973 | 0.81098 | 19 | 29 | + | TCTGTAAACCT |


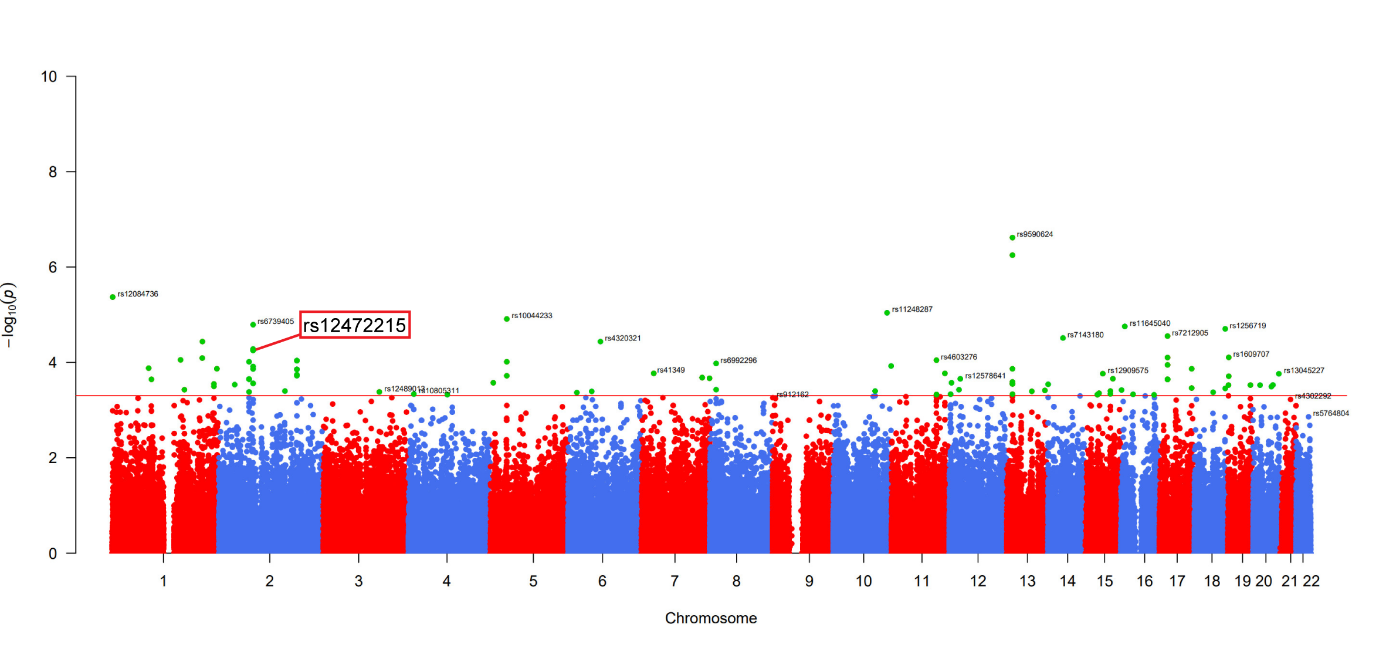


**Supplementary Figure 1.** Manhatton plot of differentially distributed SNPs between PND and non-PND elderly patients undergoing on-pump cardiacsurgery revealed by GWAS. rs12472215 locates on p2 as indicated by a red square.

**Supplementary Figure 2.** Sanger sequencing for SH-SY5Y cell line on its rs12472215 position. Image shown is in reverse complement sequence. Subjc: human genome control. Query: the sequence for SH-SY5Y. Red box indicated the position of rs12472215.


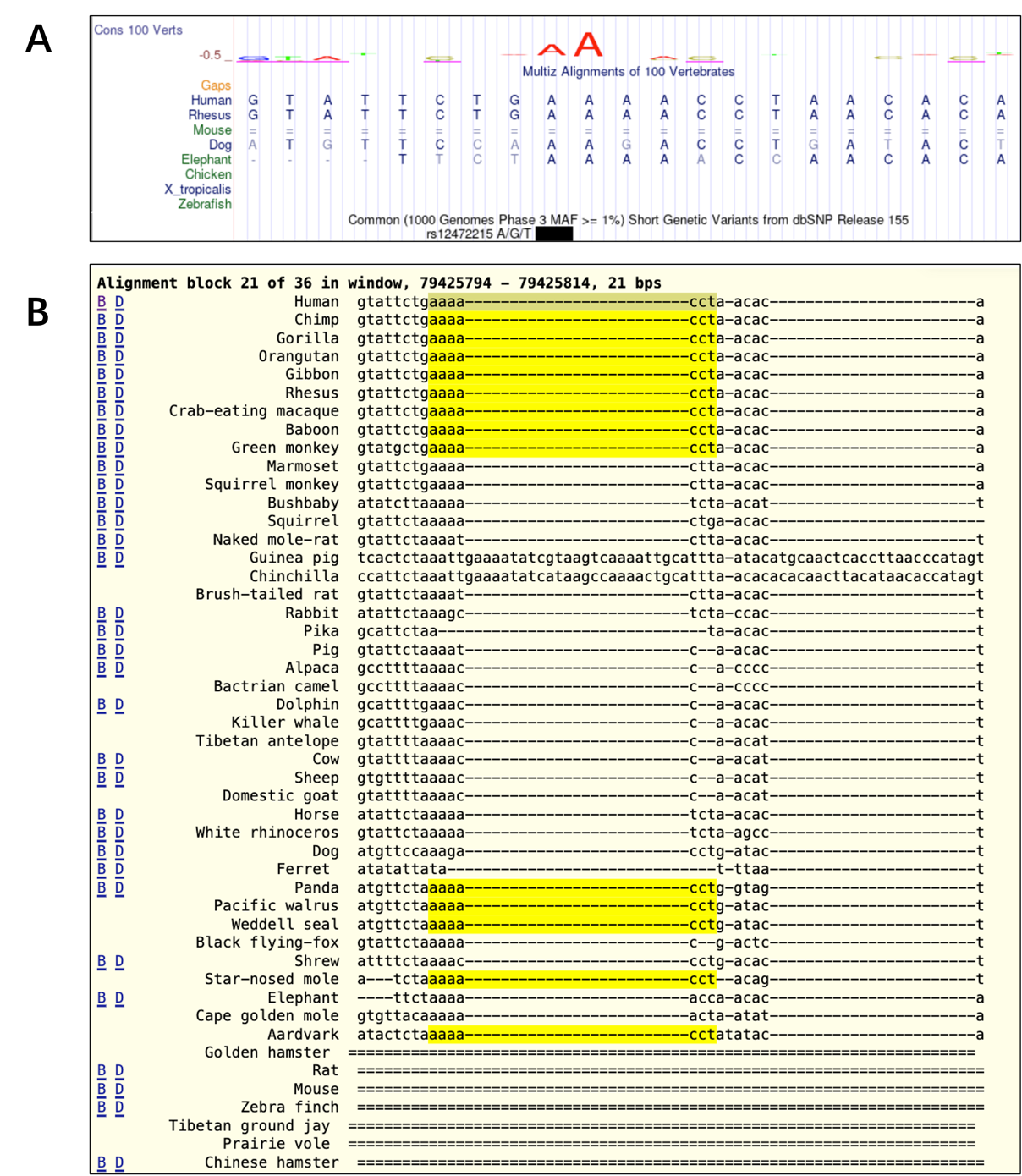


**Supplementary Figure 3.** Comparison of gene conservation for the core sequence that Foxo3 acts upon *ctnna2* near rs12472215 (chr2: 79425802) on vertebrates using Multiz Alignments of 100 Vertebrates. A. Multiz Alignments of 100 Vertebrates from chr2:79425794-79425814. B. Alignment of block showing that the human sequence is conserved in primates. Image generated from http://genome.ucsc.edu/.
