# Supplementary material for: A Decoy Oligodeoxynucleotides Disturbing Forkhead Box O3 Mediated ctnna2 Transcriptional Repression Prevents Postoperative Neurocognitive Disorder in Mice
Source: CNS Neurosci Ther. 2025 Aug 26;31(8):e70454. doi: 10.1111/cns.70454 (PMC12380581; doi:10.1111/cns.70454)

Full blot for **Figure2-Q** α-N-catenin, Marker (Proteintech, PL00003).

Chemi Hi Sensitivity

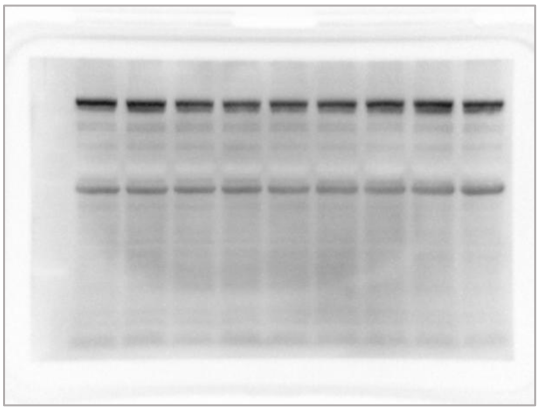

Colorimetric

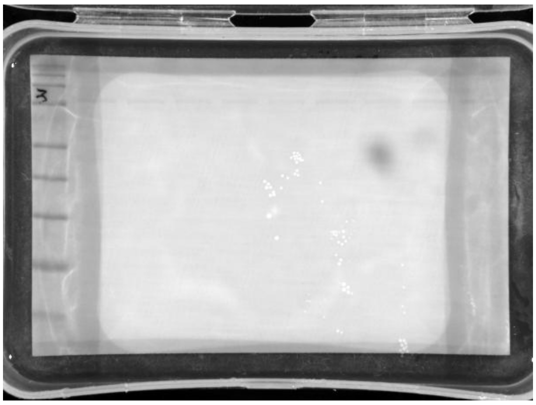

Merge

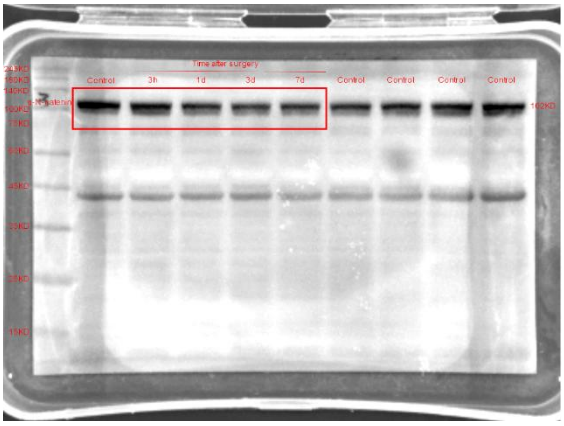

Full blot for **Figure2-Q** β-actin, Marker (Proteintech, PL00003).

Chemi Hi Sensitivity

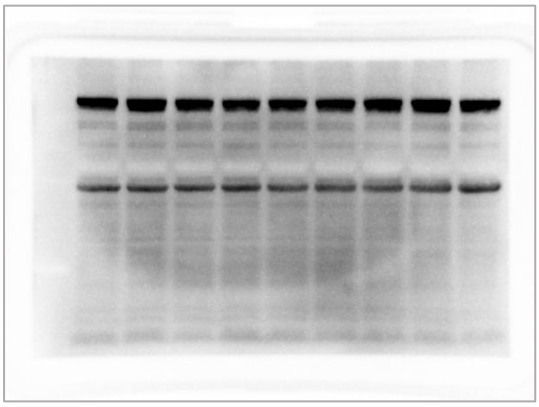

Colorimetric

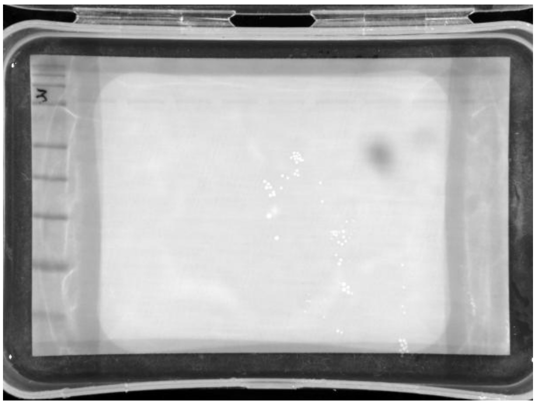

Merge

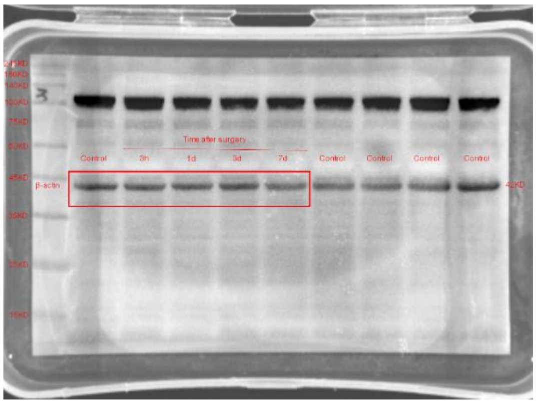

Full blot for **Figure3-B** α-N-catenin &β-actin, Marker (Thermo Fisher, 26616)).

Chemi Hi Sensitivity

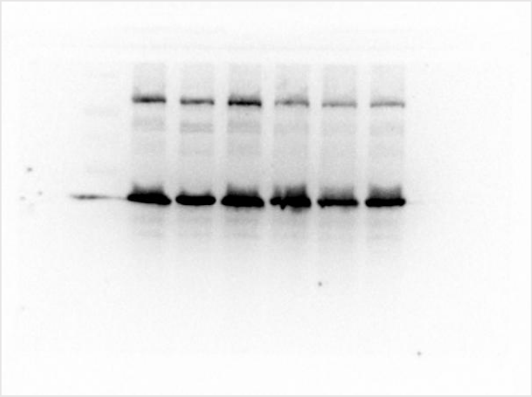

Colorimetric

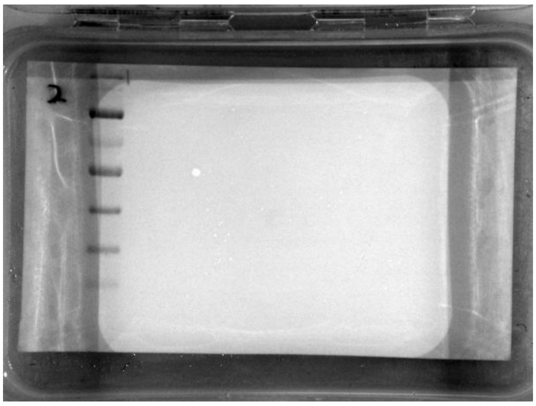

Merge

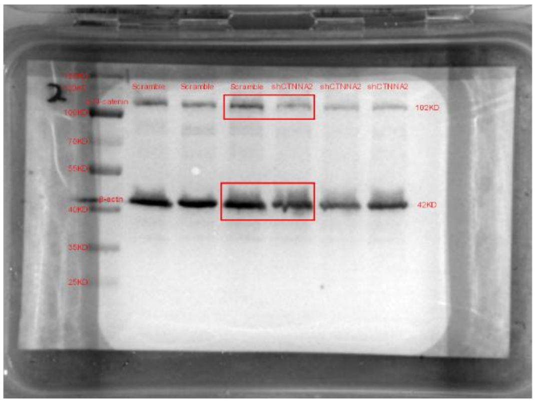

Full blot for **Figure3-J** α-N-catenin, Marker (Proteintech, PL00001).

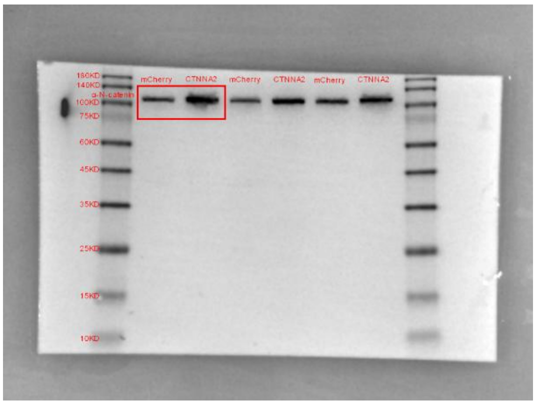

Full blot for **Figure3-J** β-actin, Marker (Proteintech, PL00001).

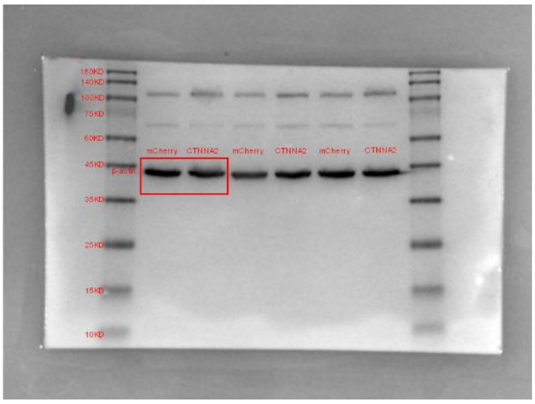

Full blot for **Figure4-B** Foxo3, Marker (Thermo Fisher, 26616)).

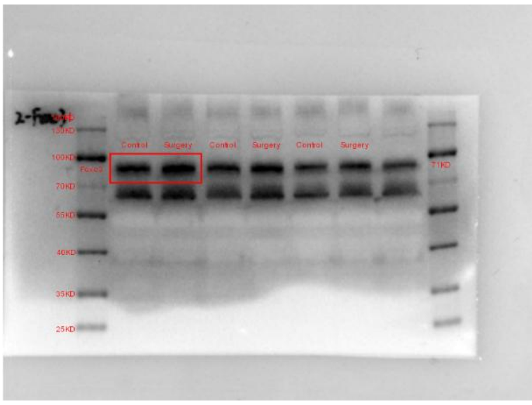

Full blot for **Figure4-B** β-actin, Marker (Thermo Fisher, 26616)).

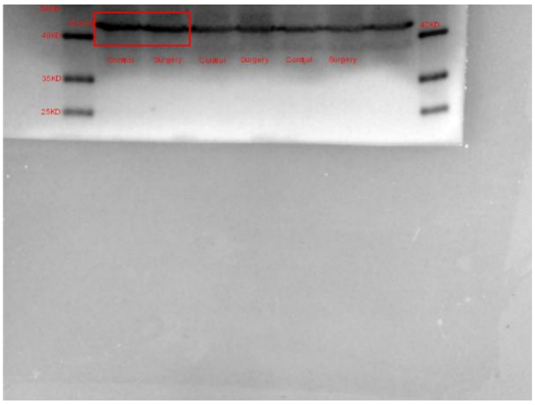

Full blot for **Figure4-C** α-N-catenin, Marker (Proteintech, PL00001).

Chemi Hi Sensitivity

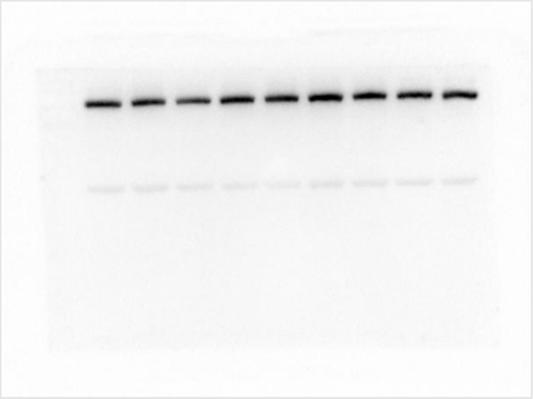

Colorimetric

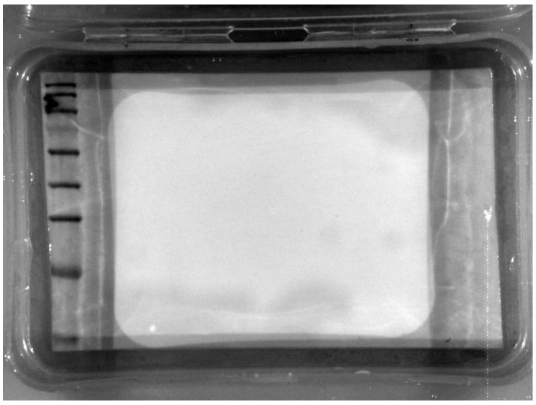

Merge

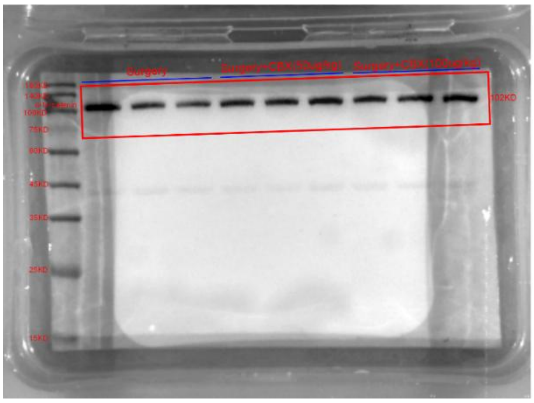

Full blot for **Figure4-C** β-actin, , Marker (Proteintech, PL00001).

Chemi Hi Sensitivity

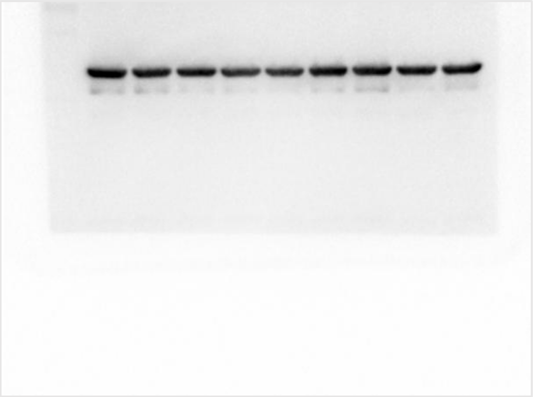

Colorimetric

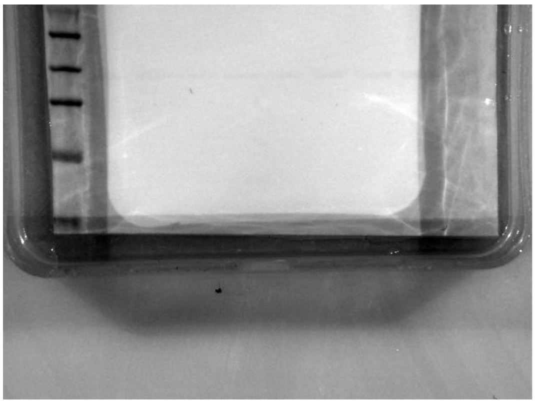

Merge

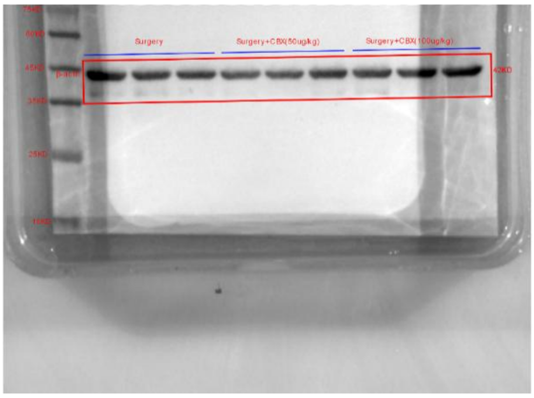

Full blot for **Figure4-E** α-N-catenin, Marker (Proteintech, PL00001).

Chemi Hi Sensitivity

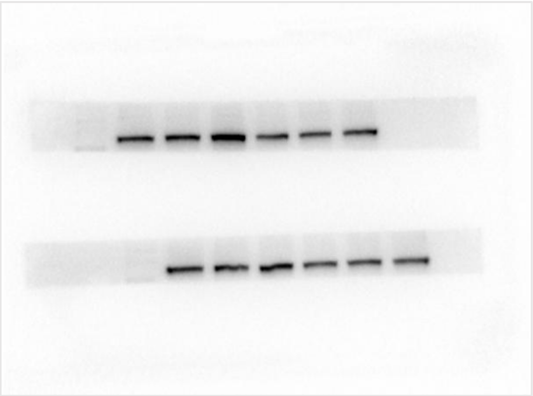

Colorimetric

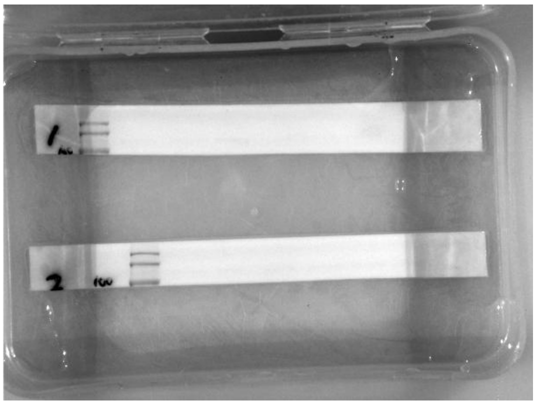

Merge

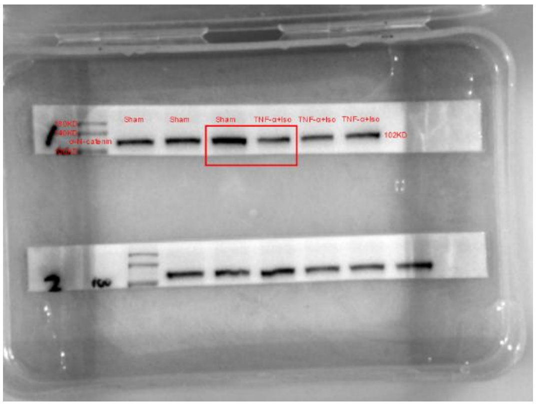

Full blot for **Figure4-E** β-actin, Marker (Proteintech, PL00001).

Chemi Hi Sensitivity

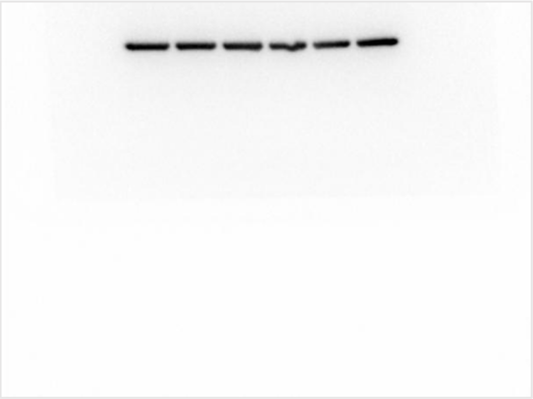

Colorimetric

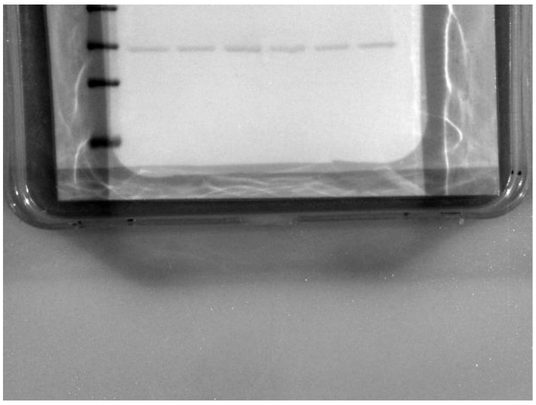

Merge

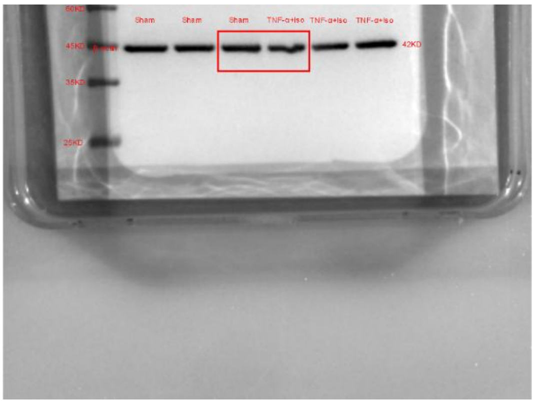

Full blot for **Figure4-M** ASCL1 (IP: Mouse anti-Foxo3), Marker (Proteintech, PL00001).

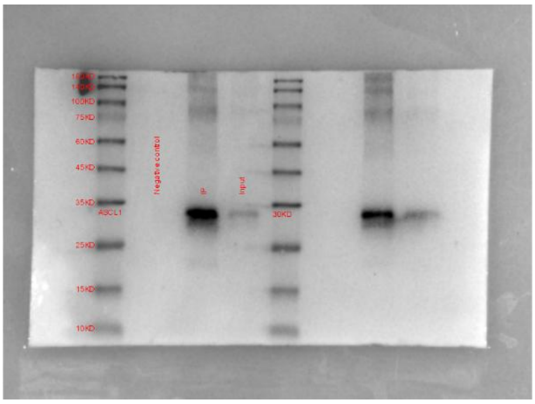

Full blot for **Figure4-M** Foxo3, Marker (Proteintech, PL00001).

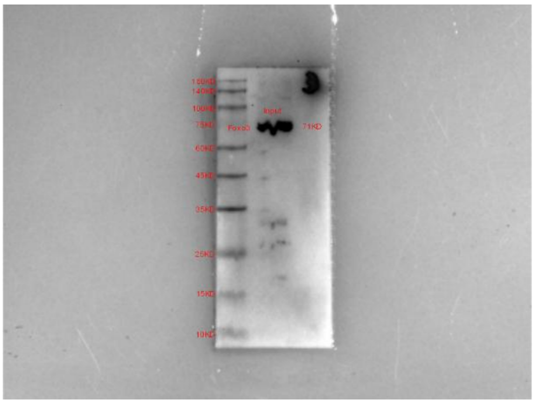

Full blot for **Figure4-N** α-N-catenin, Marker (Thermo Fisher, 26616)).

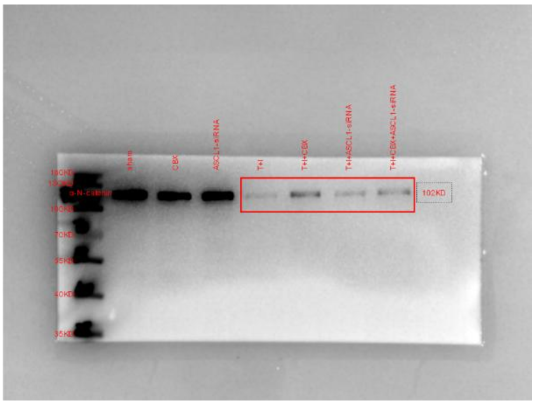

Full blot for **Figure4-N** β-tubulin, Marker (Thermo Fisher, 26616)).

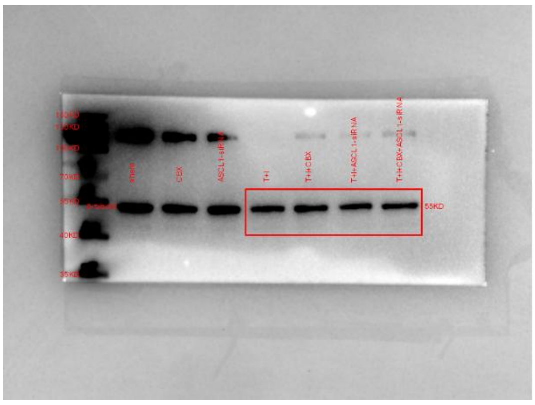

Full blot for **Figure5-A** SirT1, Marker (Thermo Fisher, 26616)).

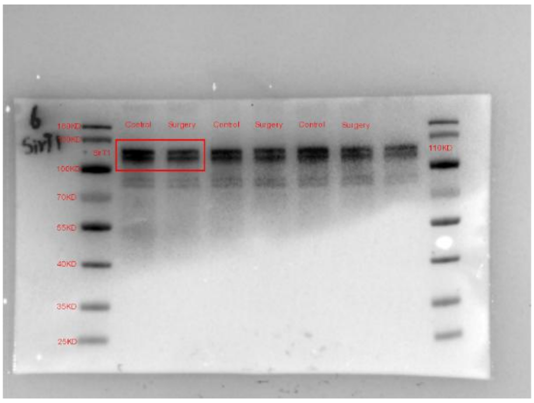

Full blot for **Figure5-A** β-actin, Marker (Thermo Fisher, 26616)).

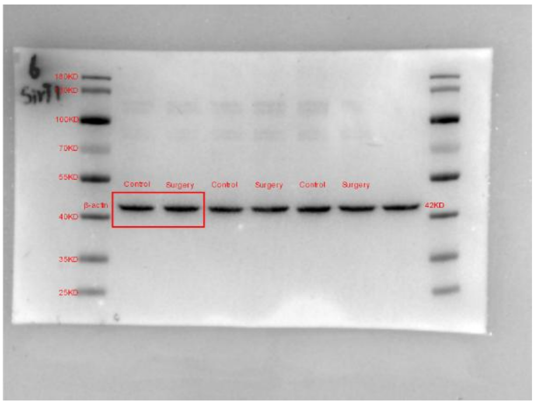

Full blot for **Figure5-B** Acetyl-foxo3, Marker (Thermo Fisher, 26616)).

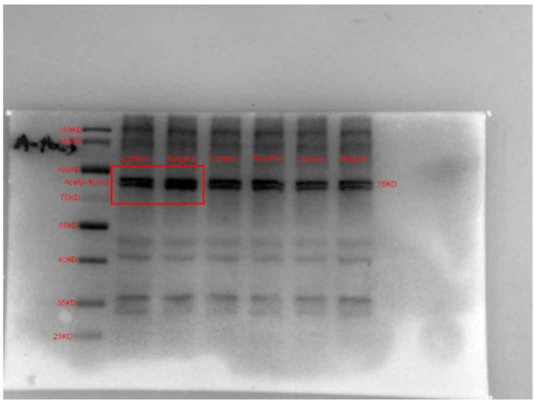

Full blot for **Figure5-B** β-actin, Marker (Thermo Fisher, 26616)).

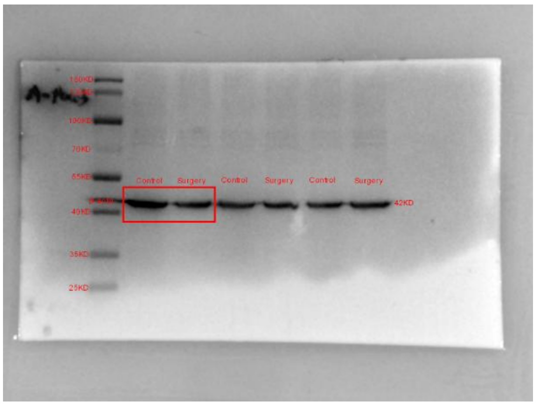

Full blot for **Figure5-C** SirT1, Marker (Thermo Fisher, 26616)).

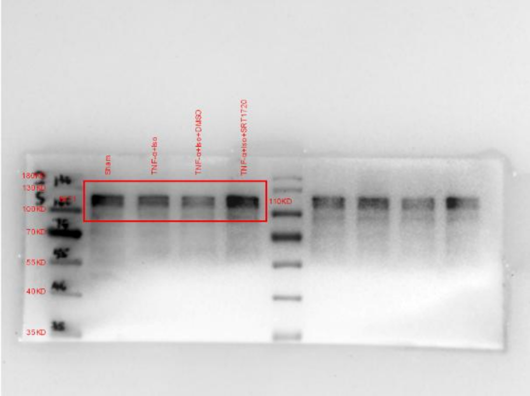

Full blot for **Figure5-C** β-tubulin, Marker (Thermo Fisher, 26616)).

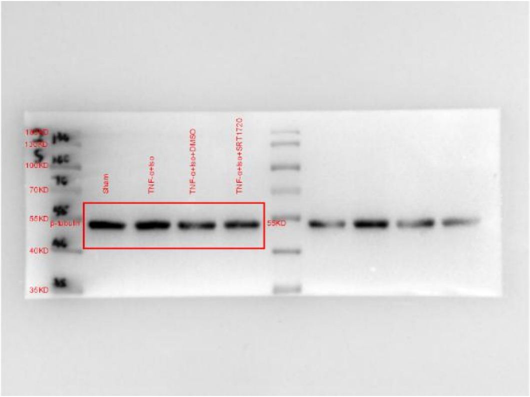

Full blot for **Figure5-D** Acetyl-foxo3, Marker (Thermo Fisher, 26616)).

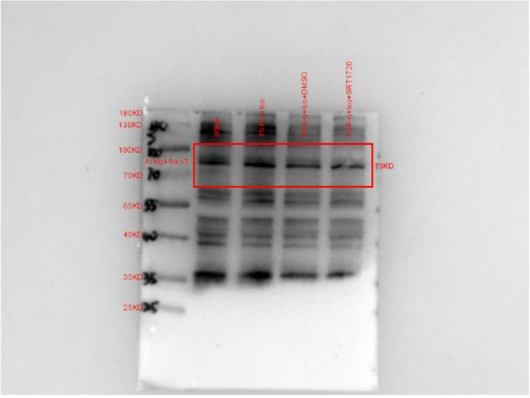

Full blot for **Figure5-D** β-tubulin, Marker (Thermo Fisher, 26616)).

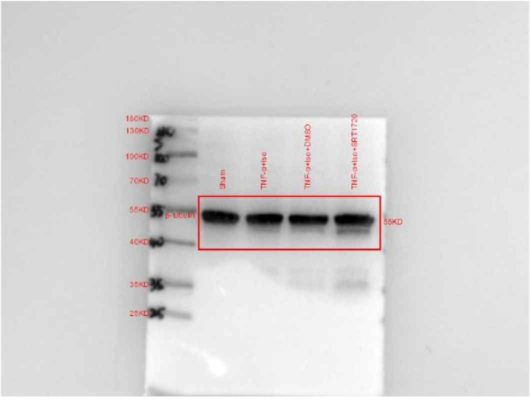

Full blot for **Figure5-E** α-N-catenin, Marker (Thermo Fisher, 26616).

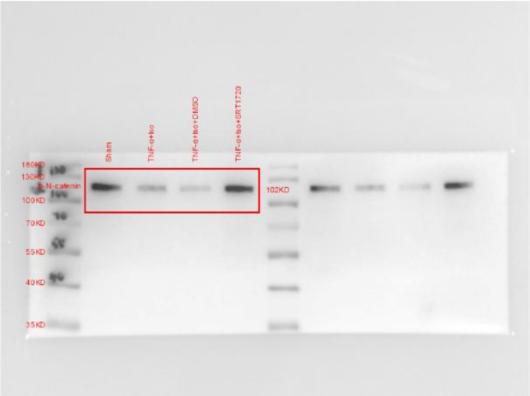

Full blot for **Figure5-E** β-tubulin, Marker (Thermo Fisher, 26616).

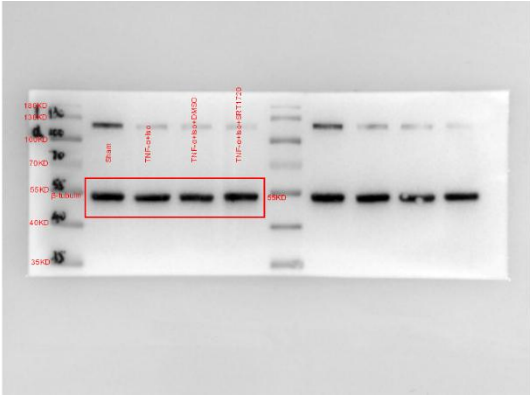

Full blot for **Figure6-C** α-N-catenin, Marker (Proteintech, PL00001).

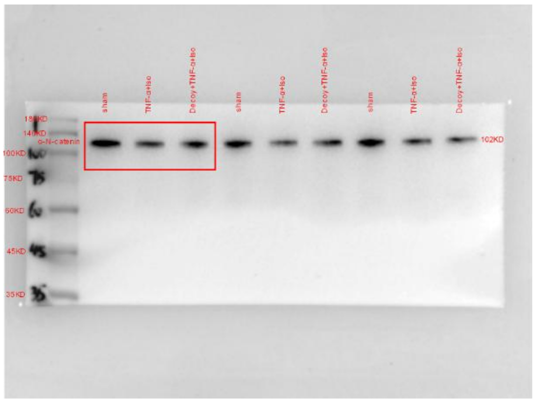

Full blot for **Figure6-C** β-tubulin, Marker (Proteintech, PL00001).

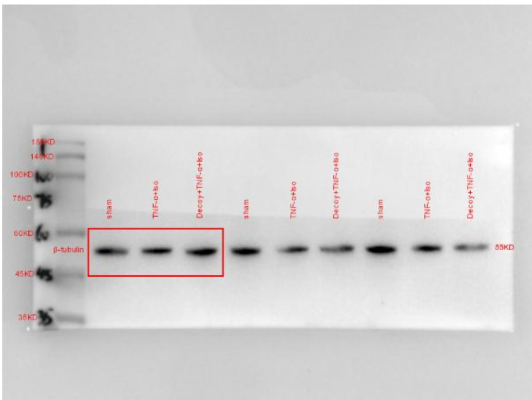

Full blot for **Figure6-D** α-N-catenin, Marker (Thermo Fisher, 26616).

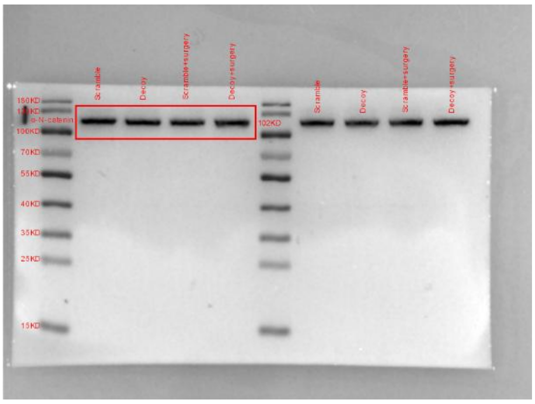

Full blot for **Figure6-D** β-tubulin, Marker (Thermo Fisher, 26616).

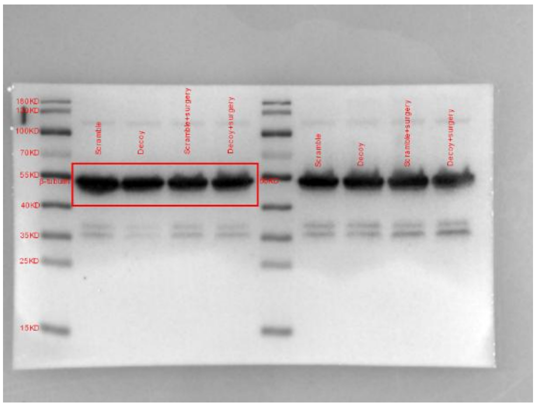

Marker used in this experiment are showed as bellow

Marker (Proteintech, PL00001)

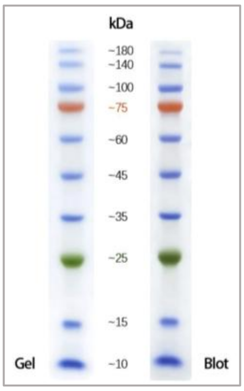

Marker (Proteintech PL00003)

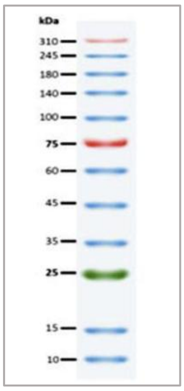

Marker (Thermo Fisher,26616)

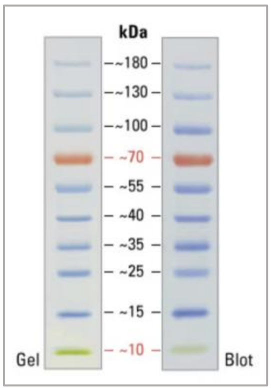

Supplement: Supplementary file 2 — Appendix S2. [file CNS-31-e70454-s001.pdf]
